# Learning to See Peaks: Attention-Based Feature Extraction for Automated Chromatographic Peak Detection

**DOI:** 10.1021/acsomega.6c01862

**Published:** 2026-05-27

**Authors:** Daniel Walter, Mathias Helbig, Birgit Weydanz, Dominik Voltmer, Juan Jose Bonfiglio, Carsten Marr, Tobias Großkopf

**Affiliations:** † Pharma Research and Early Development, 43564 Roche Diagnostics GmbH, Penzberg 82377 Germany; ‡ Department of Medicine III, 9150 Ludwig-Maximilian-University Hospital, Marchioninistr. 15, Munich 81377, Germany; § Institute of AI for Health, Computational Health Center, Helmholtz Zentrum München, Ingolstädter Landstrasse 1, Neuherberg 85764, Germany; ∥ German Cancer Consortium (DKTK), partner site Munich, a partnership between DKFZ and LMU University Hospital Munich, Heidelberg 69120 Germany; ⊥ Munich Center for Machine Learning (MCML), Munich 80538 Germany

## Abstract

Reliable peak detection remains a bottleneck in size-exclusion chromatography (SEC) as overlapping signals, drifting baselines, and analyst variability limit reproducibility. As SEC is a routine release and comparability assay and its interpretation depends on peak morphology and context, machine learning methods are well-suited to improve reproducibility at scale. We present the Peak Feature Extractor 1 (PFE-1), a one-dimensional encoder-only transformer trained on millions of synthetic chromatograms generated by a simulator statistically calibrated to routine SEC data from antibodies and related large-molecule species. PFE-1 outputs probabilistic region and event predictions that are aggregated through a transparent rule-based procedure into interpretable peak boxes. We evaluate PFE-1 on synthetic benchmarks and on a curated real SEC benchmark, reporting window-level precision/recall/F1 and box-level agreement via an intensity-weighted box loss aligned with routine process annotations. Across these evaluations, PFE-1 outperforms convolutional and derivative-based baselines, with the largest gains observed under more challenging overlap and morphology conditions. On synthetic data, PFE-1 achieves substantially higher box-level agreement than both baselines; on the curated real SEC benchmark, it likewise achieves the strongest box-level agreement while requiring no sample-specific inputs (e.g., expected peak windows). We provide a reproducible and extensible SEC-specific framework for chromatographic peak detection that supports a more consistent peak interpretation in routine analytical workflows.

## Graphical abstract

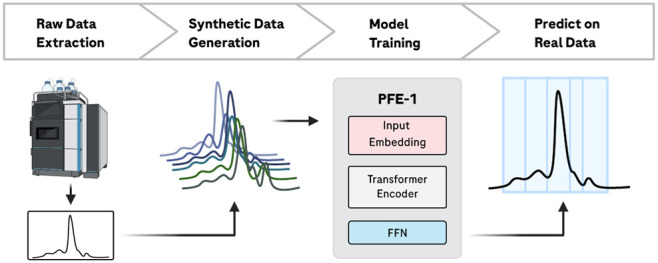

## Introduction

Size-exclusion chromatography (SEC) is a cornerstone of biopharmaceutical analytics for monitoring antibody integrity and aggregation.^1,2^ Despite robust automation of data acquisition, chromatogram interpretation remains the rate-limiting step.^3^ Overlapping signals,^4–6^ drifting baselines,^7–10^ and analyst-dependent integration decisions continue to constrain throughput and reproducibility, particularly in high-volume screening and comparability studies.^11–13^ Automated, reproducible interpretation is therefore essential for scalable analytical development.

Historically, chromatographic peak detection has relied on rule-based procedures.^14^ Derivative filters with Savitzky–Golay smoothing and curve-fitting approaches are transparent, fast, and easily audited, but they implicitly assume stable baselines, symmetric peak shapes, and the absence of tailing or fronting effects.^15^ Under drift, elevated noise, or partial overlap, thresholds and assumed peak counts become brittle, leading to missed or merged peaks.^16–18^ Learning-based methods, particularly convolutional neural networks (CNNs), improve tolerance to noise by learning peak morphologies directly from data.^19^ However, SEC peak shape and noise characteristics vary with product, matrix, and run conditions. CNNs may generalize poorly when test morphologies deviate from training data, and their local receptive fields lack the global context required to resolve shoulders and complex overlaps.^20,21^ A context-aware approach that remains consistent with laboratory integration practice is still lacking.

Transformer architectures have become state-of-the-art in sequence and vision modeling because multihead self-attention captures long-range dependencies.^22–24^ Their reach now extends to analytical chemistry, enabling component resolution in gas chromatography–mass spectrometry (GC–MS),^25^ peak quantification in liquid chromatography–mass spectrometry (LC–MS) metabolomics,^26^ and peak assignment in nuclear magnetic resonance (NMR) spectroscopy.^27^ This ability to resolve overlapping patterns makes transformers particularly suitable for SEC, yet architectures optimized for chromatographic conventions have not been systematically evaluated (Figure 1).

**Figure 1:**
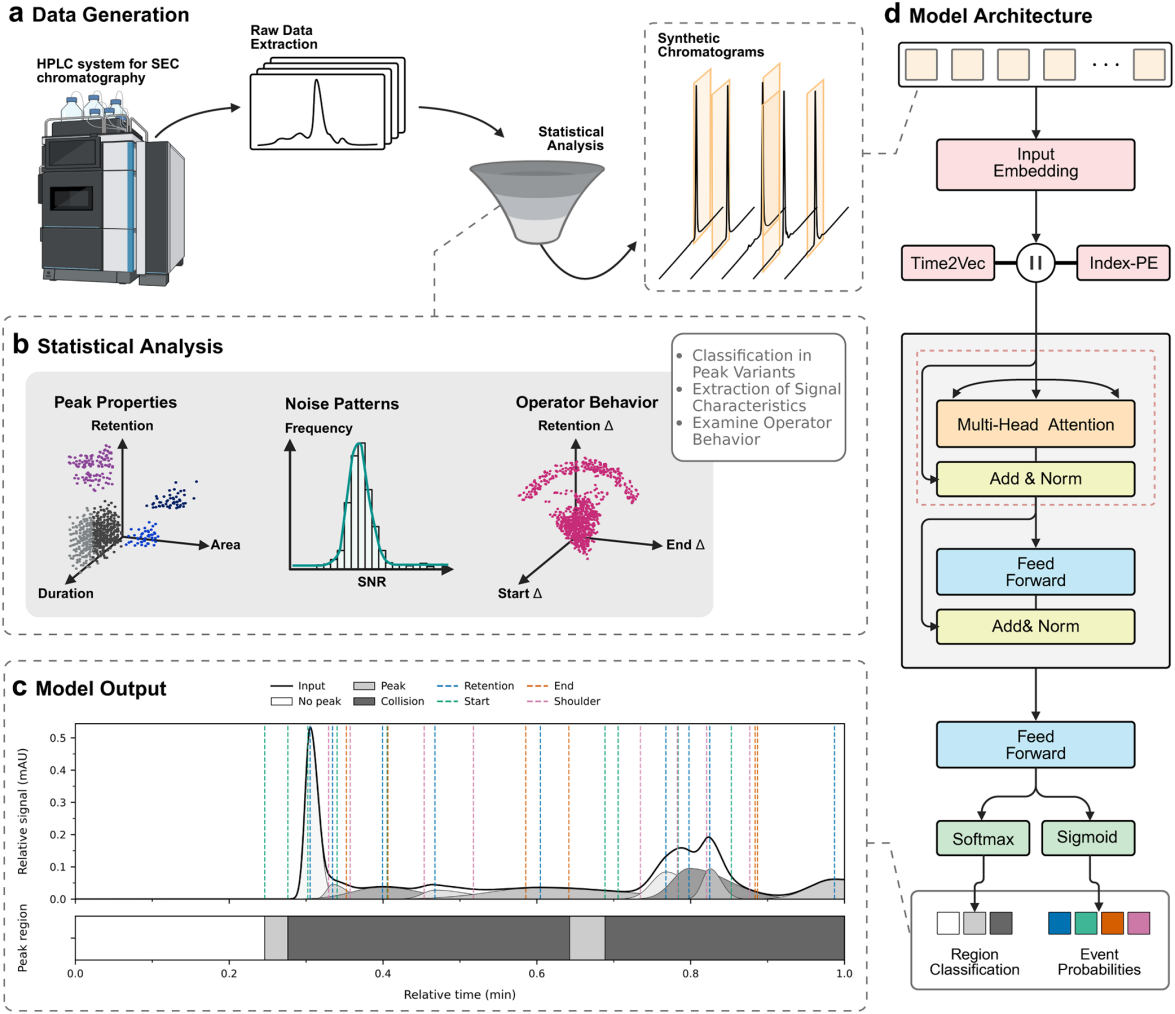
Workflow for data generation, statistical analysis, and transformer-based modeling of chromatographic peak detection. (a) Raw SEC chromatograms from high-performance liquid chromatography systems are profiled to capture peak and noise behavior, then augmented into overlapping synthetic windows for training. (b) Statistical summaries of peak properties, noise patterns, and operator behavior parametrize the simulator and contextualize routine variability. (c) Representative model output showing the input signal (black), predicted events (vertical guides), expected component peaks (gray), and three region labels (No peak, Peak, Collision). (d) Architecture of PFE-1: each window receives concatenated Time2Vec and index encodings, then passes through a transformer encoder; the schematic style and color scheme in (d) follow Vaswani et al.^32^ before yielding softmax-based region classes plus sigmoid event probabilities. Created in BioRender. Walter, D. (2026) https://BioRender.com/svcz9ik.

While large quantities of SEC data are available, labeling heterogeneity and inconsistent annotations limit their use for supervised learning. These data stem from anonymized routine SEC measurements collected across multiple products and methods at a biopharmaceutical development site. Among approximately 648,000 chromatograms, fewer than one-sixth (104,000) possess reliable event labels across all peak types. To overcome this limitation, we developed a statistical simulator calibrated to experimental SEC distributions. Synthetic chromatograms generated by this simulator formed the foundation for training the transformer-based peak-detection model.

In SEC practice, analysts apply vertical-line (VL) integration, defining each peak by start, retention (apex), and end and using local minima as boundaries in overlaps.^28,29^ This convention has long been established and remains embedded in most chromatography data systems and routine laboratory workflows. To reflect this workflow, we introduce a boxlike intersection-over-union (IoU)-based metric tailored to vertical-line (VL) boxes.^30^ The formulation includes adjustable error weighting and normalization across peak sizes, making it suitable for a box-level evaluation. On synthetic data with complete event information, we complement box-level reporting with cross-entropy-based event- and region-level metrics.

To address the limitations above, we present Peak Feature Extractor 1 (PFE-1), a one-dimensional, encoder-only transformer for SEC chromatograms. PFE-1 uses multihead self-attention to model long-range dependencies and outputs region labels (nonpeak, peak, collision) and event probabilities (start, retention, end, shoulder). Peak boxes are then assembled by transparent rules; the position is represented via a temporal Time2Vec embedding.^31^ This feature-first design provides the global context needed to separate partially overlapping signals while remaining aligned with the routine SEC review. Trained on millions of synthetic chromatograms, PFE-1 improved box-level agreement over the evaluated baseline algorithms across the reported synthetic and curated SEC benchmarks.

## Materials and Methods

### Data Preprocessing

We analyzed 648,000 anonymized routine SEC chromatograms collected at the Roche Innovation Center Munich (RICM) between 2021 and 2024. The corpus originates from release and comparability assays for antibodies and related large-molecule species, including antibody-derived fusion and multispecific formats. Data were acquired on validated ultrahigh-performance liquid chromatography (UHPLC) platforms equipped with autosamplers, thermostated column compartments, and ultraviolet–visible (UV–Vis) detectors operating at 280 nm. Run times spanned roughly 2 to 60 min, covering the method diversity summarized in Figure 1a. Throughout this manuscript, a “trace” refers to one UV-280 nm readout of a single sample injection.

Systematic quality control yielded 104,000 chromatograms with consistent metadata and verified annotations. Records containing nonstandard labels were excluded to avoid bias during simulator calibration and benchmarking. Expert annotations originating from routine vertical-line (VL) practice were mapped onto a harmonized label space comprising (i) region classes that encode the number of simultaneously active peaks (0 = baseline, 1 = single-peak region, 2 = collision region) and (ii) event markers (start, retention, end, and shoulder) aligned to the resampled temporal grid.

Native detector rates of 4–10 Hz were resampled to 1 Hz using linear interpolation. Negative intensities were clipped to zero, and each trace was min–max normalized to the [0,1] interval. For downstream benchmarking, we curated an expert-reviewed subset of 412 traces representing analytically relevant, morphology-rich cases with high agreement between routine annotations and secondary review. “Complex” here denotes traces containing overlapping structures, shoulders, or multiple minor species in addition to the main peak, whereas “high-consensus” denotes traces for which expert review confirmed the routine annotation as sufficiently stable for comparative benchmarking. Longer chromatograms were cropped to study-relevant integration segments to mirror batch-review practice and to focus the task on method-relevant peak interpretation rather than on wash or nontarget regions. All records were anonymized, and sensitive manufacturing details were removed; dataset-level diagnostics and the project-level composition of the curated benchmark appear in Texts S1.1–S1.3, Table S1.1, and Figures S1.1–S1.3.

Real-world chromatograms served exclusively to calibrate the statistical simulator and estimate the empirical peak/noise distributions. Model training used synthetic signals only, whereas benchmarking relied on an independent collection of experimental SEC traces, ensuring a strict separation among calibration, training, and evaluation. These preprocessing steps yielded the harmonized corpus that underpins the following simulations and model training.

### Simulator

The simulator concept and parametrization are illustrated in Figure 1a,b. To generate fully labeled training data at scale while controlling signal complexity and removing operator variability, we synthesized fixed-length chromatographic windows that emulate routine SEC measurements after 1 Hz resampling. Each synthetic signal is expressed as the sum of asymmetric peaks following a split-normal distribution with separate left and right standard deviations, enabling tailing or fronting shapes consistent with experimental observations.^33^ Peak amplitudes, centers, asymmetries, and interpeak distances were drawn from bounded ranges derived from the routine corpus (see Text S2.1, Text S2.2, Table S2.1, and Figure S2.1 for calibrated ranges; Texts S3.1–S3.4 detail the split-normal construction, overlap handling, and window synthesis).

Signals were perturbed in two stages to emulate instrument variability: (i) a short-term noise process sampled from white, Gaussian, uniform, and green noise families at small amplitudes, and (ii) a low-order baseline component implemented as a constant offset or shallow quadratic drift with magnitudes sampled from empirical ranges. We extended traces beyond the target window and applied random cropping to enrich boundary conditions and avoid alignment artifacts. For each window, the simulator outputs the intensity vector plus region labels and event markers, enabling probabilistic supervision and downstream rule-based aggregation. Texts S3.2–S3.4 formalize the sampling logic, boundary adjustments, and event labeling. The canonical noise profiles are depicted in Figure S2.2, illustrating the family-specific variability across representative amplitudes.

All distributions governing peak shape, signal-to-noise ratio, interpeak distance, and drift magnitude were calibrated from the quality-controlled chromatograms. Random number generators were deterministically seeded to ensure reproducibility while enabling a massive synthesis. This statistically grounded simulator bridges experimental variability and model training, ensuring that the synthetic corpus reproduces the distributions visualized in Figure 1B.

### Model and Training

Figure 1c,d outlines the PFE-1 architecture. The model maps each chromatographic window to two aligned outputs per time point: a three-class region distribution (baseline, single peak, collision) and four event probabilities (start, retention, end, shoulder). Scalar intensities are linearly projected into a higher-dimensional embedding and concatenated with two complementary positional cues: an index-based embedding and a *Time2Vec* embedding that adds sinusoidal components to encode recurrent retention time patterns while preserving interpretability.^31^ The network employs a moderate-depth, encoder-only transformer with multihead self-attention, residual connections, and dropout confined to attention paths. A shared, lightweight feed-forward projection maps encoder states to the region and event heads, enabling joint representation learning while decoupling calibration of the two output types. Ancillary architectural details (class weights, dropout placement, serialization) are compiled in Text S4.1.

Models were trained solely on synthetic windows using AdamW^34^ with a step-decay learning-rate schedule and early stopping on validation loss. Stratified sampling across simulator regimes ensured balanced coverage of morphologies. Reproducibility was enforced through explicit seeding across frameworks, deterministic cuDNN settings, and fixed dataloader seeds. For benchmarking, we reimplemented a one-dimensional CNN baseline aligned with Kensert et al.^21^ and a derivative-based Savitzky–Golay (SG) detector. The CNN contains substantially fewer parameters than PFE-1, reflecting its role as a lightweight literature baseline rather than a capacity-matched comparator. Both baselines were trained on identical labels and evaluated with the same suite to ensure comparability. Hyperparameters and architectural factors were explored through multistage Sobol-sampled searches followed by targeted ablations and robustness experiments; the resulting corridors, fixed-grid scans, and seed-variance statistics appear in Texts S5.1–S5.3. Runtime characteristics, throughput traces, and hardware provisioning are reported in Texts S4.2 and S4.3.

### Aggregation to Peak Boxes and Evaluation

The conversion of model outputs into interpretable peak intervals follows standard vertical line (VL) integration and is visualized in Figure 1c,d. The model itself operates on fixed windows of 250 resampled points, whereas complete chromatograms of arbitrary duration are processed through overlapping-window inference. For full chromatograms, overlapping-window predictions were merged along the time axis using a transparent rule-based pipeline (default stride = 1 s) combined with Hann tapering to suppress boundary artifacts and reduce edge discontinuities.^35^ Aligned region and event outputs were converted into deterministic peak boxes via a transparent rule-based pipeline: contiguous high-probability regions defined candidate intervals, local maxima in event logits identified start, end, retention, and shoulder positions, and logical consistency checks enforced valid ordering while pruning ultrashort or contradictory intervals. Unless stated otherwise, all reported results used this deterministic aggregation.

Agreement between predicted and reference peak intervals was quantified with a normalized, intensity-weighted Box-Loss score (higher is better). Predicted and ground-truth boxes were first paired via greedy intersection-over-union (IoU) matching. For scoring, each temporal interval [*s*, *e*] was converted into a signal-derived rectangle *B* = (*x*_1_, *y*_1_, *x*_2_, *y*_2_) with inclusive bounds *x*_1_ = *s*, *x*_2_ = *e*, *y*_1_ = min_*t*__∈__[*s*,*e*]_ *x*_*t*_, and *y*_2_ = max_*t* ∈ [*s*,*e*]_ *x*_*t*_, where *x*_*t*_ denotes the normalized intensity trace at time index *t*. Matched peaks were then scored using the standard intersection-over-union for two rectangles *U* and *V*,

1 $$\mathrm{IoU}\left(U,V\right)=\frac{\left|U\cap V\right|}{\left|U\right|+\left|V\right|-\left|U\cap V\right|}$$

where |·| denotes rectangle area. For each chromatogram *n*, we compute a raw mismatch penalty:

2 $$P_{\mathrm{box}}^{\left(n\right)}=\lambda_{\mathrm{TP}}P_{\mathrm{TP}}^{\left(n\right)}+\lambda_{\mathrm{FN}}P_{\mathrm{FN}}^{\left(n\right)}+\lambda_{\mathrm{FP}}P_{\mathrm{FP}}^{\left(n\right)}$$

3 $$P_{\mathrm{TP}}^{\left(n\right)}=\underset{i\in \mathrm{TP}}{\sum }\left(1-\mathrm{IoU}\left(B_{i}^{\mathrm{ref}},B_{i}^{\mathrm{pred}}\right)\right)w\left(B_{i}^{\mathrm{ref}}\right)$$

4 $$P_{\mathrm{FN}}^{\left(n\right)}=\underset{j\in \mathrm{FN}}{\sum }w\left(B_{j}^{\mathrm{ref}}\right)$$

5 $$P_{\mathrm{FP}}^{\left(n\right)}=\underset{k\in \mathrm{FP}}{\sum }w\left(B_{k}^{\mathrm{pred}}\right)$$

where TP, FN, and FP denote true-positive, false-negative, and false-positive boxes, respectively. The scalars λ_TP_, λ_FN_, λ_FP_ allow fine-grained control over how each class influences the total penalty. Over an evaluation set *D*, penalties are aggregated as $P_{\mathrm{box}}\left(D\right)=\underset{n\in D}{\sum }P_{\mathrm{box}}^{\left(n\right)}$ . For normalization, we compute a dataset-specific reference penalty *P*_max_(*D*) by evaluating the same weighted TP/FN/FP terms after replacing each model prediction with one full-span interval $B_{\mathrm{full}}^{\left(n\right)}=\left\{\left(0,T_{n}\right)\right\}$ , where *T*_*n*_ denotes the last valid time index of chromatogram *n*. We then report the normalized score,

6 $$L_{\mathrm{box},\mathrm{norm}}\left(D\right)=\frac{P_{\max}\left(D\right)-P_{\mathrm{box}}\left(D\right)}{P_{\max}\left(D\right)}$$

so values closer to 1 indicate better agreement. Unless stated otherwise, “Box-Loss” denotes the normalized score *L*_box,norm_ (higher is better). Normalization and weighting details are given in Text S5.4; worked example conversions from region/event outputs to peak boxes, including representative failure modes, are shown in Text S6.1 and Figures S6.1–S6.14.

Model performance was assessed at two complementary levels: window-level accuracy on synthetic benchmarks using precision, recall, and F_1_ for region classification and event detection, and box-level agreement using the normalized Box-Loss for both simulated and experimental chromatograms. Statistical uncertainty was summarized by 95% bootstrap confidence intervals (CIs); multiple comparisons used Holm correction, and effect sizes were reported via nonparametric measures. Hyperparameter search logs were further analyzed with tree-based surrogate models to identify promising regions and salient interactions. To contextualize automation relative to current practice, we additionally conducted a reader study with trained analysts using the vertical-line (VL) workflow. “Analyst consensus” refers to the mean Box-Loss between each analyst annotation and the routine process annotation across the 15 reader-study chromatograms.

## Results and Discussion

### Hyperparameter Optimization and Model Evaluation

PFE-1 was optimized to balance performance, efficiency, and transferability to experimental SEC data. The final configuration (Text S4.1) uses eight encoder layers, eight attention heads, an embedding width of 256, hidden/feed-forward dimensions of 2048, and dropout of 0.2 applied solely within attention paths. Training employed AdamW with a base learning rate of 1 × 10^–4^, weight decay of 0.01, a step size of ten epochs, a decay factor γ = 0.05, and gradient accumulation to reach an effective global batch size of roughly 496 across three million synthetic windows. Training pipeline details and runtime diagnostics are reported in Text S4.2 and Table S4.1.

Contour plots expose the narrow corridor that delivered stable convergence: learning rate versus weight decay for different dataset sizes (Figure S5.1), embedding versus feed-forward dimensions (Figure S5.2), and encoder depth versus attention heads (Figure S5.3). A combined fixed-grid summary (Figure S5.4) then compares attention-head count, dataset scale, and encoder depth directly, revealing diminishing returns and variance growth beyond the selected configuration. The Sobol contours, fixed-grid diagnostics, and seed-sensitivity summaries are detailed in Texts S5.1–S5.3, confirming that event-level F_1_ shows the largest random-seed variation while region-level and box metrics remain tightly clustered.

Analogous to Guo et al.^25^ and Zhang et al.,^26^ the reported configuration relies on AdamW with step-decay scheduling. The combined fixed-grid sweeps show that scaling the dataset beyond three million windows yields only marginal gains, while increasing model capacity beyond the published range amplifies variance without producing commensurate improvements within the stable operating corridor (Figure S5.4). The additional capacity sweeps in Text S5.10 further show that, within the PFE family, performance on synthetic data approaches a plateau once the model reaches the low tens-of-millions parameter range, both in validation loss and in Box-Loss. The most robust configurations lie in the range above roughly 20 million parameters, placing the selected configuration in the broad scale of ResNet-50 rather than at an arbitrary point in parameter space.^36^ Within the scope of this study, model selection therefore prioritized the most robust synthetic performance within this plateau regime rather than maximal parameter efficiency or a claim of global architectural optimality.

### Ablation Studies

Beyond the multiphase Sobol search, we performed controlled ablations in which a fixed reference configuration was held constant while one factor at a time varied. Figure 1c anchors these factors: the encoding components correspond to the input projection layer, whereas the encoder activation resides within the gray feed-forward module. Table 1 summarizes the main outcomes.

**Table 1:** Ablation Study on Encoding and Activation Factors Relative to the Reference PFE-1 Configuration^a^

Factor	Levels compared	ΔEvent-F_1_	ΔRegion-F_1_	Main effect *F*(*p*)	Post hoc comparison (adjusted *p*)	Cliff’s δ
Encoding type	Time2Vec vs Index	+0.06	+0.09	262 (1 × 10^–25^)	Time2Vec > Index (<0.001)	0.73 (large)
Encoding method	Concat vs Add	+0.02	+0.03	1533 (3 × 10^–50^)	Concat > Add (0.01)	0.55 (med.)
Encoder activation	GELU vs ReLU	+0.04	+0.05	–(*p* < 0.01)	GELU > ReLU (0.009)	0.62 (large)
Encoder activation	GELU vs SiLU	+0.03	+0.04	–(*p* ≈ 0.008)	GELU > SiLU (0.008)	0.45 (med.)
Output activation	ReLU vs GELU	n.s.	n.s.	n.s.	–	–

a Reported metrics summarize main and post hoc effects.

#### Encoding Strategy

Incorporating Time2Vec delivered the largest gain, significantly improving both event- and region-level metrics relative to fixed index-based encodings. Concatenating encodings rather than adding them yielded an additional yet moderate benefit. Factorial ANOVA confirmed a dominant main effect of Time2Vec on both metrics (Event-F_1_: *F* ≈ 262, *p* ≈ 1 × 10^–25^; Region-F_1_: *F* ≈ 1533, *p* ≈ 3 × 10^–50^), with interactions primarily affecting Region-F_1_.

#### Activation Functions

Among the nonlinearities tested, GELU consistently outperformed ReLU and SiLU within the encoder, producing the highest F_1_ scores for both event and region classification, whereas the output activation had no measurable effect. Two-way ANOVA confirmed a significant main effect of the encoder activation for both endpoints; output activation and interaction terms were nonsignificant. Holm-corrected Mann–Whitney tests indicated large effects for GELU > ReLU and moderate effects for GELU > SiLU.

### Benchmark Results on Synthetic Data

The statistically calibrated simulator supplies fully specified labels, enabling granular comparisons. Under these conditions, box-level agreement clearly separates the three approaches (Figure 2a): the derivative-based Savitzky–Golay (SG) detector reaches a normalized Box-Loss of 0.339 ± 0.026, the CNN baseline reaches 0.842 ± 0.007, and PFE-1 reaches 0.953 ± 0.008 (higher is better). Thus, PFE-1 remains the strongest model on the synthetic benchmark, while the CNN baseline substantially outperforms Savitzky–Golay (SG). The weak derivative performance is expected because shifting noise profiles and heterogeneous peak morphologies cause it to recover mainly the most prominent peaks.

**Figure 2:**
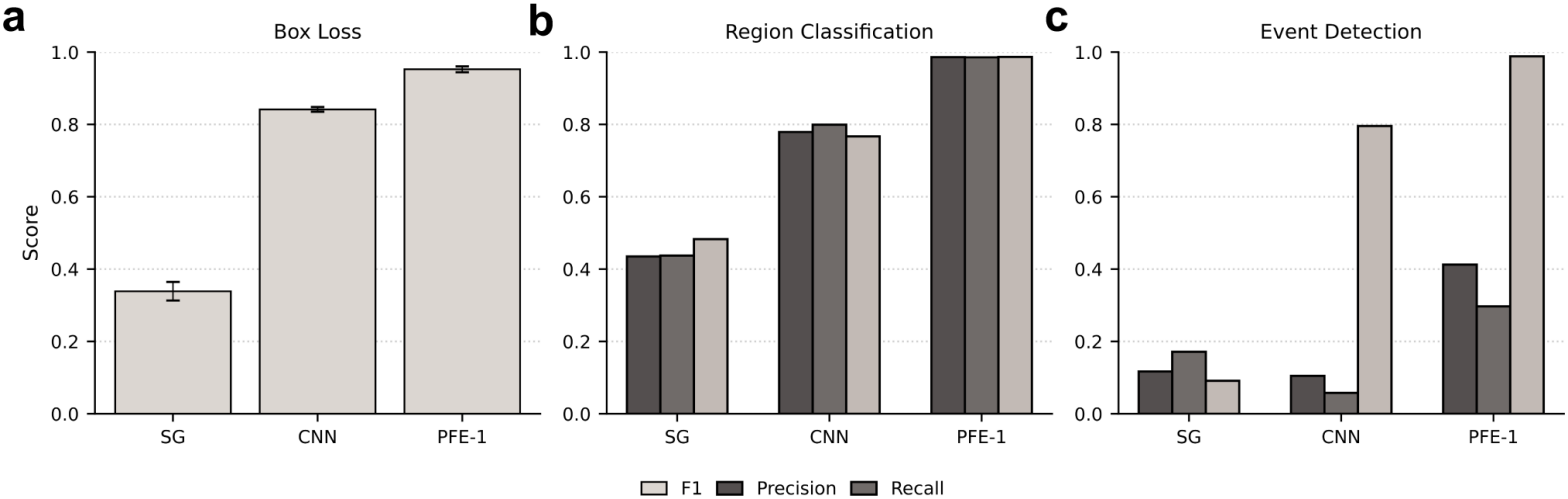
Performance comparison on synthetic data. (a) Box-Loss (higher = better; normalized score as defined in Methods) for the derivative-based baseline (Savitzky–Golay (SG)), the CNN baseline, and PFE-1 on synthetic chromatograms. PFE-1 shows the highest agreement with simulator-derived ground truth, followed by the CNN baseline and Savitzky–Golay (SG). Error bars denote the standard deviation across replicate evaluations. (b) Region classification metrics for Savitzky–Golay (SG), CNN, and PFE-1. (c) Event detection metrics for Savitzky–Golay (SG), CNN, and PFE-1. Bars show F1, precision, and recall; PFE-1 achieves the strongest overall region- and event-level performance, while the CNN baseline shows markedly higher event recall than precision. Created in BioRender. Walter, D. (2026) https://BioRender.com/3y6b0oi.

At the window level (Figure 2b,c), PFE-1 delivers near-perfect region classification (F_1_ = 0.986, precision = 0.986, recall = 0.987), clearly outperforming both the CNN baseline (F_1_ = 0.779) and Savitzky–Golay (SG) (F_1_ = 0.435). Event detection follows the same overall hierarchy: PFE-1 yields the strongest aggregate event F_1_ (0.412) and the highest event recall (0.988), while the CNN baseline remains strongly recall-biased (precision = 0.058, recall = 0.796, F_1_ = 0.105). Savitzky–Golay (SG) performs worst overall on both box- and region-level synthetic metrics and attains only modest event-level agreement (event F_1_ = 0.117). These patterns are consistent with the qualitative prediction examples in Figure 3: PFE-1 produces sharper, more internally consistent region and event outputs, whereas CNN tends to overactivate and Savitzky–Golay (SG) misses many weaker or interacting structures.

**Figure 3:**
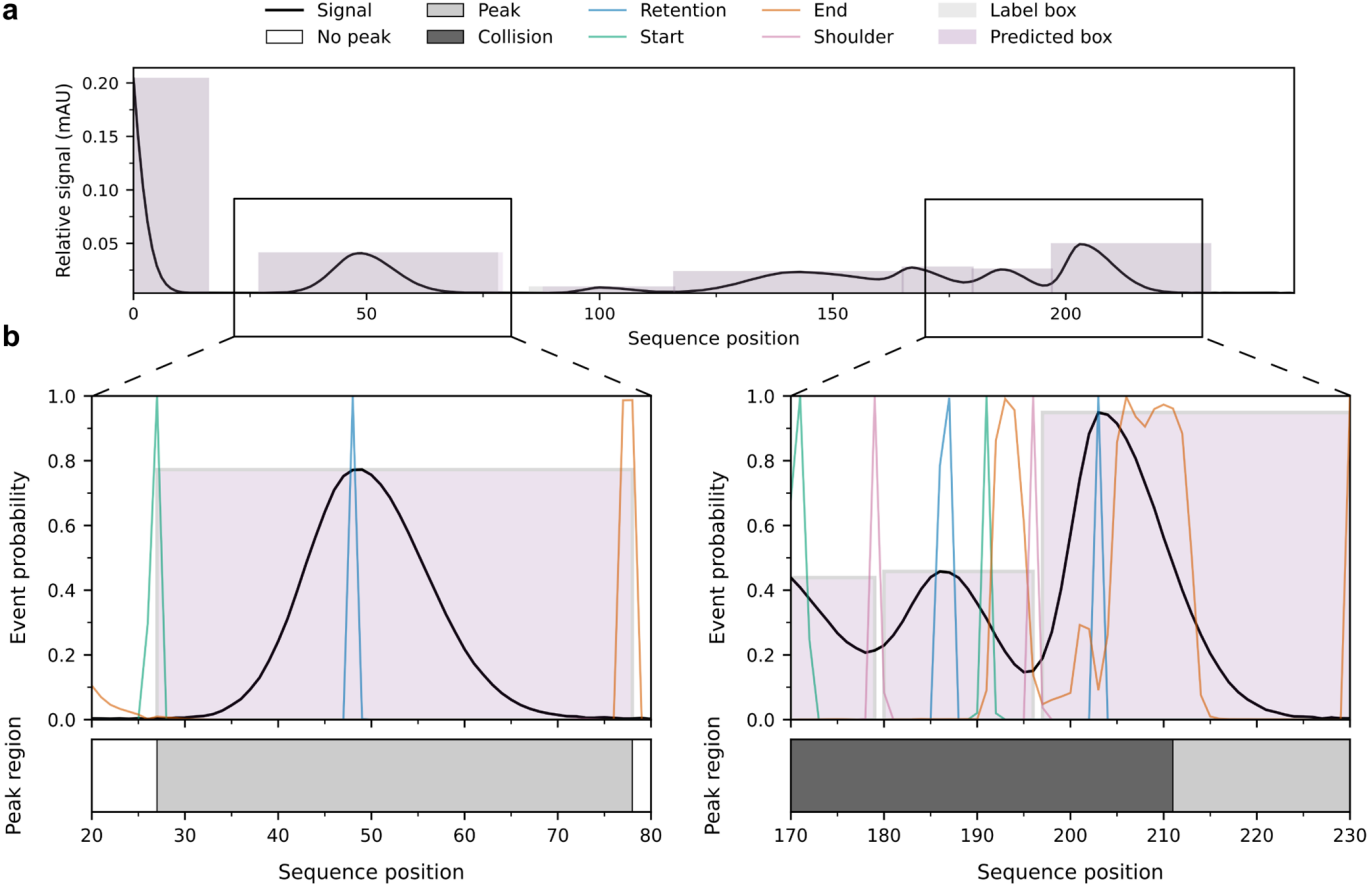
Representative model predictions on synthetic chromatograms. (a) Example prediction for a synthetic SEC trace showing the input signal (black, relative signal in mAU), with ground truth (gray) and model-predicted (purple) peak boxes. The predicted intervals closely align with the reference annotations, exhibiting only minor boundary deviations, particularly for isolated peaks. (b) Zoomed views of two representative regions. Left: a single, well-resolved peak where the predicted start and end events delineate a valid box confirmed by the retention (apex) event. The region classification is clean, and the event probabilities are sharp and well separated, facilitating straightforward postprocessing. Right: a collision region containing multiple overlapping peaks. Here, the model maintains stable predictions; shoulder events (pink) are used as boundary markers to form boxes consistent with vertical-line (VL) integration practice and the behavior of human analysts. Created in BioRender. Walter, D. (2026) https://BioRender.com/tdlssau.

Qualitatively, PFE-1 produces crisp event distributions and stable region probabilities that separate baseline, single-peak, and collision states (Figure 3). The CNN baseline, in contrast, yields diffuse activations with overlapping responses, which complicate downstream aggregation. These differences matter in practice: PFE-1 outputs align naturally with rule-based postprocessing.

Error analysis on the synthetic set revealed characteristic patterns. Because the simulator represents traces as overlapping split-normal peaks, multiple plausible decompositions can exist; congested regions, therefore, produce broader, less specific probability masses and occasional interference. Window-boundary artifacts appear when true events fall just outside the crop, inducing spurious edge activations; representative edge effects and box-conversion examples are shown in Text S6.1 and Figures S6.1–S6.14. Coactivations between event classes (e.g., shoulder/start) arise in dense mixtures but are largely resolved by the aggregation rules.

### Benchmark Results on Real Data

To evaluate transfer to experimental SEC traces, window-level predictions were aggregated into peak boxes using the same deterministic rules. Performance was compared against routine process annotations on the curated real SEC benchmark via the normalized, intensity-weighted Box-Loss. Worked example conversions on real chromatograms are provided in Text S7.1 and Figures S7.1–S7.6. As contextual information on human variability, we additionally conducted a reader study in which 12 analysts from different departments independently annotated 15 SEC chromatograms with method-typical peak windows supplied. Although peak counts differed for every sample, integrated areas were highly consistent and tracked the process annotations closely. Analyst consensus, computed as the mean Box-Loss between each analyst and the process annotation, reached 0.99 ± 0.01.

Because full-trace inference requires overlap-based reconstruction, absolute box scores depend in part on the stride, merge, and solver settings. On GENA, these reconstruction choices produce the characteristic nonmonotonic dependence summarized in Text S5.9. Unless otherwise noted, all reported results use a stride of 1 s.

Figure 4 shows a representative challenging example from GENA (normalized Box-Loss: 0.8580; see Text S7.1). The example illustrates similarity to human integration behavior at the level of contextual grouping, for example, in the unsplit structure around position 150, while also showing the model’s tendency toward oversegmentation and occasional broad, low-amplitude boxes that would not be annotated as peaks by a human analyst.

**Figure 4:**
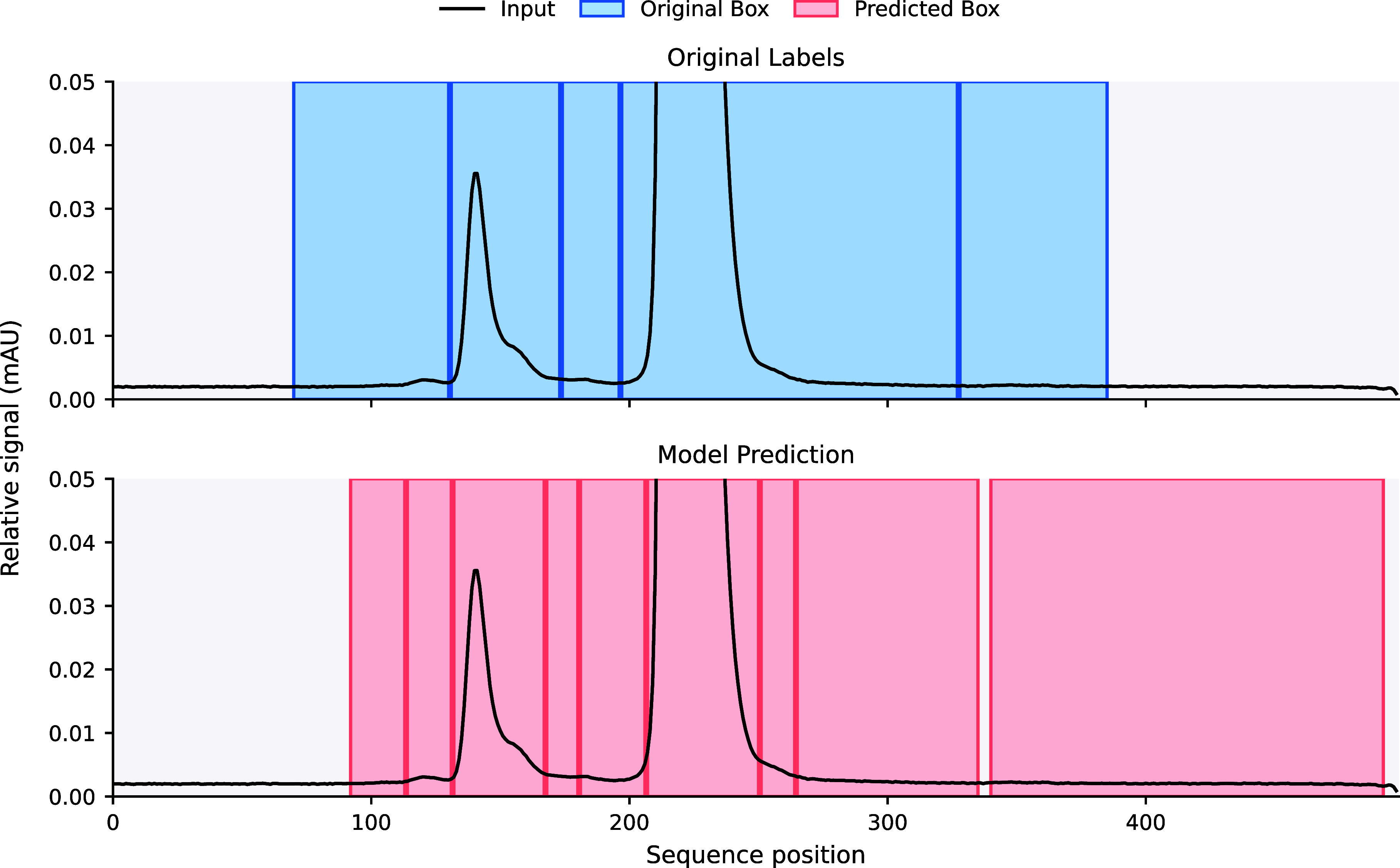
Representative negative example from the curated GENA benchmark. The signal is shown on a relative-intensity scale of 0–0.05 to make low-amplitude structures visible despite the large dynamic range. Top: routine reference annotation with peak boxes shown in blue. Bottom: PFE-1 prediction under the same postprocessing pipeline used for benchmarking. The example attains a normalized Box-Loss of 0.8580 and illustrates both context-aware grouping and oversegmentation in low-amplitude regions; detailed box-level calculations are provided in Text S7.1.

On the curated real benchmark, PFE-1 achieved a normalized Box-Loss of 0.928 ± 0.003, outperforming both baselines (CNN: 0.893 ± 0.004; Savitzky–Golay (SG): 0.873 ± 0.005; Figure 5). Thus, PFE-1 remained the strongest model on this benchmark, while the CNN baseline ranked above Savitzky–Golay (SG). The smaller gap between the learned baselines on real than on synthetic data underscores that transfer depends not only on architecture but also on how closely the synthetic training regime, annotation style, and reconstruction heuristics align with the evaluated real-data setting. The curated benchmark is dominated by low-noise chromatograms with a pronounced main peak and weaker satellites, a regime that remains analytically relevant for routine byproduct review but does not exhaust the full range of SEC difficulty. Additional worked examples and box-level calculations are provided in Text S7.1, and distributional differences between synthetic and real windows are summarized in Text S1.3.

**Figure 5:**
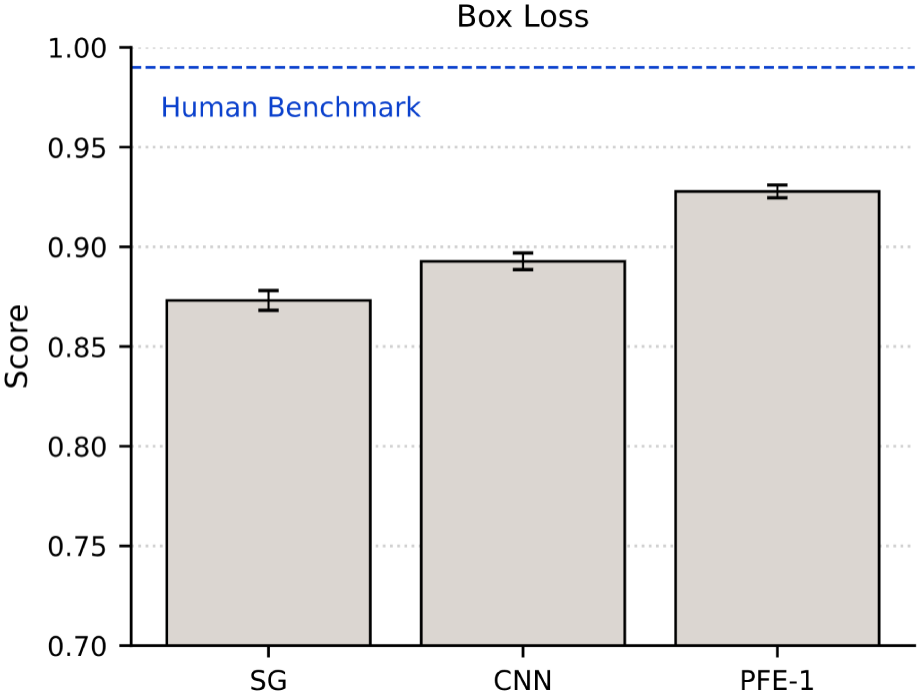
Performance comparison on real-size exclusion chromatography data. Box-Loss (higher = better; normalized score as defined in Methods) for the derivative-based baseline (Savitzky–Golay (SG)), the CNN baseline, and PFE-1 on the curated real SEC benchmark. Error bars denote the standard deviation across replicate evaluations. The dashed line indicates the analyst consensus, computed as the mean Box-Loss between individual analyst annotations and the routine process annotation, and is shown as a contextual reference rather than as the primary evaluation target. PFE-1 achieves the highest box-level agreement on this benchmark, followed by the CNN baseline and Savitzky–Golay (SG). Created in BioRender. Walter, D. (2026) https://BioRender.com/s59u66c.

Failure modes on experimental traces mirror synthetic observations: occasional coactivations of shoulder/start events in congested regions and rare boundary artifacts when true events lie just outside a window. Both effects are largely mitigated by center-weighted merging and post hoc consistency checks.

### Advantages and Limitations

Compared with non-deep-learning methods such as the derivative baseline, PFE-1 attains higher agreement with simulator-derived reference labels and the strongest box-level agreement on the curated real SEC benchmark. Unlike many software-integrated tools, PFE-1 requires no sample-specific inputs (e.g., expected peak windows) and can be applied across varying peak counts and trace lengths through a window-based prediction. Its probabilistic outputs remain interpretable and support the transparent aggregation into boxes. Relative to the CNN baseline, PFE-1 combines stronger region-level consistency with better event localization on synthetic data and retains the highest box-level agreement after being transferred to the curated real benchmark.

These gains involve trade-offs. PFE-1’s parameter count and compute footprint exceed those of the baselines, necessitating greater hardware and training time (runtime analyses are recorded in Text S4.2). Full-trace predictions rely on a rule-based postprocessing pipeline that is not trained end-to-end with the encoder, introducing a potential mismatch between training and evaluation objectives. Robustness analyses in Texts S5.5–S5.9 show that reasonable changes in Box-Loss formulation and reconstruction settings shift absolute scores, but the main model ranking remains stable across the tested variants. Although the synthetic training distribution is statistically calibrated, it remains an approximation of routine conditions. Additional comparisons between synthetic and real windows (Text S1.3, Figure S1.3) reveal structured differences in signal-to-noise ratio, peak width, and label fragmentation, indicating a measurable but nonuniform domain shift; the curated real benchmark therefore represents a practically relevant SEC regime rather than exhaustive routine coverage. Finally, performance was strongest within a relatively narrow corridor of capacity, learning rate, and regularization.

To mitigate these trade-offs, we emphasize compact operating points. Standard efficiency techniques—knowledge distillation, pruning, quantization—and tighter coupling between encoder and aggregation (e.g., differentiable or jointly trained postprocessing) are natural future directions. Targeted fine-tuning and simulator enrichment also hold promise. Prospective evaluation on broader and less curated routine SEC datasets, including regimes with stronger noise, denser overlap, and more heterogeneous annotation patterns, will be important to establish the scope of generalization beyond the benchmark studied here.

## Supplementary Material



## Notes

Additional preprocessing diagnostics (Texts S1.1–S1.3; Figures S1.1–S1.3), simulator calibration and generation details (Texts S2.1–S3.4; Figures S2.1–S2.2; Table S2.1), model/training and reproducibility diagnostics (Texts S4.1–S5.10; Figures S5.1–S5.12; Table S4.1), and worked box-conversion examples on synthetic and real chromatograms (Texts S6.1–S7.1; Figures S6.1–S7.6) (PDF)

## References

[1] D’Atri V., Imiołek M., Quinn C., Finny A., Lauber M., Fekete S., Guillarme D. (2024). Size exclusion chromatography of
biopharmaceutical products: From current practices for proteins to
emerging trends for viral vectors, nucleic acids and lipid nanoparticles. J. Chromatogr. A.

[2] Brusotti G., Calleri E., Colombo R., Massolini G., Rinaldi F., Temporini C. (2018). Advances on Size Exclusion Chromatography
and Applications on the Analysis of Protein Biopharmaceuticals and
Protein Aggregates: A Mini Review. Chromatographia.

[3] Wu K.-W., Chen T.-H., Yang T.-C., Wang S.-C., Shameem M., Graham K. S. (2023). Continuous monitoring
of a monoclonal antibody by size
exclusion chromatography reveals a correlation between system suitability
parameters and column aging. J. Pharm. Biomed.
Anal..

[4] Mondello L., Cordero C., Janssen H.-G., Synovec R. E., Zoccali M., Tranchida P. Q. (2025). Comprehensive
two-dimensional gas chromatography–mass
spectrometry. Nat. Rev. Methods Primers.

[5] Stefanuto P.-H., Smolinska A., Focant J.-F. (2021). Advanced chemometric
and data handling
tools for GC × GC-TOF-MS. TrAC, Trends
Anal. Chem..

[6] Pérez-Cova M., Jaumot J., Tauler R. (2021). Untangling
comprehensive two-dimensional
liquid chromatography data sets using regions of interest and multivariate
curve resolution approaches. TrAC, Trends Anal.
Chem..

[7] Yi L., Dong N., Yun Y., Deng B., Ren D., Liu S., Liang Y. (2016). Chemometric
methods in data processing of mass spectrometry-based
metabolomics: A review. Anal. Chim. Acta.

[8] Asnin L. D. (2016). Peak measurement
and calibration in chromatographic analysis. TrAC, Trends Anal. Chem..

[9] Lopatka M., Barcaru A., Sjerps M. J., Vivó-Truyols G. (2016). Leveraging
probabilistic peak detection to estimate baseline drift in complex
chromatographic samples. J. Chromatogr. A.

[10] Parastar H., Christmann J., Weller P. (2024). Automated 2D peak detection in gas
chromatography-ion mobility spectrometry through persistent homology. Anal. Chim. Acta.

[11] Haidar
Ahmad I. A. (2017). Necessary Analytical Skills and Knowledge for Identifying,
Understanding, and Performing HPLC Troubleshooting. Chromatographia.

[12] Satwekar A., Panda A., Nandula P., Sripada S., Govindaraj R., Rossi M. (2023). Digital by design approach to develop
a universal deep learning AI
architecture for automatic chromatographic peak integration. Biotechnol. Bioeng..

[13] Evard H., Kruve A., Leito I. (2016). Tutorial on estimating the limit
of detection using LC-MS analysis, part I: Theoretical review. Anal. Chim. Acta.

[14] Felinger A. (1998). 8 Peak detection. Data Handl.
Sci. Technol..

[15] Bos T. S., Knol W. C., Molenaar S. R., Niezen L. E., Schoenmakers P. J., Somsen G. W., Pirok B. W. (2020). Recent
applications of chemometrics
in one- and two-dimensional chromatography. J. Sep. Sci..

[16] Alam M. S., McGregor L. A., Harrison R. M. (2024). A review of organic aerosol speciation
by comprehensive two-dimensional gas chromatography. TrAC, Trends Anal. Chem..

[17] Fu H.-Y., Guo J.-W., Yu Y.-J., Li H.-D., Cui H.-P., Liu P.-P., Wang B., Wang S., Lu P. (2016). A simple multi-scale
Gaussian smoothing-based strategy for automatic chromatographic peak
extraction. J. Chromatogr. A.

[18] Zhou J., Li J., Gao W., Zhang S., Wang C., Lin J., Zhang S., Yu J., Tang K. (2022). Combination of continuous
wavelet transform and genetic algorithm-based Otsu for efficient mass
spectrometry peak detection. Biochem. Biophys.
Res. Commun..

[19] Bosten E., Kensert A., Desmet G., Cabooter D. (2024). Automated method development
in high-pressure liquid chromatography. J. Chromatogr.
A.

[20] Bueschl C., Doppler M., Varga E., Seidl B., Flasch M., Warth B., Zanghellini J. (2022). PeakBot: machine-learning-based
chromatographic
peak picking. Bioinformatics.

[21] Kensert A., Bosten E., Collaerts G., Efthymiadis K., Van Broeck P., Desmet G., Cabooter D. (2022). Convolutional
neural
network for automated peak detection in reversed-phase liquid chromatography. J. Chromatogr. A.

[22] Dosovitskiy, A. ; Beyer, L. ; Kolesnikov, A. ; Weissenborn, D. ; Zhai, X. ; Unterthiner, T. ; Dehghani, M. ; Minderer, M. ; Heigold, G. ; Gelly, S. An Image is Worth 16 × 16 Words: Transformers for Image Recognition at Scale. arxiv, 2021, 10.48550/arxiv.2010.11929.

[23] Carion, N. ; Massa, F. ; Synnaeve, G. ; Usunier, N. ; Kirillov, A. ; Zagoruyko, S. End-to-End Object Detection with Transformers. arXiv, 2020, 10.48550/arXiv.2005.12872.

[24] Strudel, R. ; Garcia, R. ; Laptev, I. ; Schmid, C. Segmenter: Transformer for Semantic Segmentation. arXiv, 2021, 10.48550/arXiv.2105.05633.

[25] Guo Z., Fan Y., Yu C., Lu H., Zhang Z. (2024). GCMSFormer: A Fully
Automatic Method for the Resolution of Overlapping Peaks in Gas Chromatography-Mass
Spectrometry. Anal. Chem..

[26] Zhang Z., Yang H., Wang Y., Zhang L., Lin S.-H. (2025). QuanFormer:
A Transformer-Based Precise Peak Detection and Quantification Tool
in LC-MS-Based Metabolomics. Anal. Chem..

[27] Zhou Z., Liao X., Qiu X., Zhang Y., Dong J., Qu X., Lin D. (2025). NMRformer:
A Transformer-Based Deep Learning Framework
for Peak Assignment in 1D ^1^H NMR Spectroscopy. Anal. Chem..

[28] Dyson, N. Chromatographic Integration Methods; The Royal Society of Chemistry, 1998; pp. 35–88.

[29] Eckerbom S., Bergqvist Y., Jeppsson J.-O. (1994). Improved Method for Analysis of Glycated
Haemoglobin by Ion Exchange Chromatography. Ann. Clin. Biochem.: Int. J. Lab. Med..

[30] Rezatofighi, H. ; Tsoi, N. ; Gwak, J. ; Sadeghian, A. ; Reid, I. ; Savarese, S. Generalized Intersection Over Union: A Metric and a Loss for Bounding Box Regression. arXiv, 2019, 10.48550/arXiv.1902.09630.

[31] Kazemi, S. M. ; Goel, R. ; Eghbali, S. ; Ramanan, J. ; Sahota, J. ; Thakur, S. ; Wu, S. ; Smyth, C. ; Poupart, P. ; Brubaker, M. Time2Vec: Learning a Vector Representation of Time. arxiv, 2019, 10.48550/arxiv.1907.05321.

[32] Vaswani, A. ; Shazeer, N. ; Parmar, N. ; Uszkoreit, J. ; Jones, L. ; Gomez, A. N. ; Kaiser, L. ; Polosukhin, I. Attention Is All You Need. arxiv, 2017, 10.48550/arxiv.1706.03762.

[33] Zou S., Cui Q., Liu J., Wu Q., Zhu L., Chen D., Du Y., Wu T. (2025). Local Asymmetric Gaussian
Fitting Algorithm for Enhanced
Peak Detection of Liquid Chromatography-High Resolution Mass Spectrometry
Data. Anal. Chem..

[34] Loshchilov, I. ; Hutter, F. Decoupled Weight Decay Regularization. arxiv, 2017, 10.48550/arxiv.1711.05101.

[35] Pielawski N., Wählby C. (2020). Introducing
Hann windows for reducing edge-effects
in patch-based image segmentation. PLoS One.

[36] He, K. ; Zhang, X. ; Ren, S. ; Sun, J. Deep Residual Learning for Image Recognition. arxiv, 2015, 10.48550/arxiv.1512.03385.

