# Supplementary material for: Learning to See Peaks: Attention-Based Feature Extraction for Automated Chromatographic Peak Detection
Source: ACS Omega. 2026 May 27;11(22):32946–54. doi: 10.1021/acsomega.6c01862 (PMC13261407; doi:10.1021/acsomega.6c01862)
Supplement: Supplementary file 1 [file ao6c01862_si_001.pdf]

Supporting Information

Learning to see peaks: Attention-based feature extraction for automated chromatographic peak detection

Daniel Walter[1], Mathias Helbig[1], Birgit Weydanz[1], Dominik Voltmer[1], Juan Jose Bonfiglio[1], Carsten Marr[2,3,4,5], Tobias Großkopf[1*]

[1]Pharma Research and Early Development, Roche Diagnostics GmbH, 82377 Penzberg, Germany

[2]Department of Medicine III, Ludwig-Maximilian-University Hospital, Marchioninistr. 15, 81377 Munich, Germany

[3]Institute of AI for Health, Computational Health Center, Helmholtz Zentrum München, Ingolstädter Landstrasse 1, 85764 Neuherberg, Germany

[4]German Cancer Consortium (DKTK), partner site Munich, a partnership between DKFZ and LMU University Hospital Munich, 69120 Heidelberg, Germany

[5]Munich Center for Machine Learning (MCML), 80538 Munich, Germany

**Corresponding author: tobias.grosskopf@roche.com*

Contents

S1. Additional details on data preprocessing (Texts S1.1–S1.3; Figs. S1.1–S1.3)

S2. Simulator calibration (Texts S2.1–S2.2; Fig. S2.1–S2.2; Tab. S2.1)

S3. Peak shape and chromatogram generation (Texts S3.1–S3.4)

S4. Model configuration, training, and hardware (Texts S4.1–S4.3; Tab. S4.1)

S5. Hyperparameter sweeps, diagnostics, and reproducibility (Texts S5.1–S5.10; Figs. S5.1–S5.12)

S6. Example conversion: model outputs to peak-box evaluation (Text S6.1; Figs. S6.1–S6.14)

S7. Example conversion on real chromatograms (Text S7.1; Figs. S7.1–S7.6)

S1 Dataset statistics

Text S1.1. Noise estimation and signal-to-noise ratio (SNR)

To characterize noise in real size-exclusion chromatography (SEC) data and to parameterize the synthetic data generator, SNR was quantified using a robust Median Absolute Deviation (MAD) approach. First-order differences were computed, $\Delta x_i = x_{i+1} - x_i$, and the noise standard deviation was estimated as

$$\sigma_{\text{noise}} = 1.4826 \cdot \text{median}\left(|\Delta x_i - \text{median}(\Delta x)|\right),$$

with the factor 1.4826 ensuring consistency with the standard deviation of normally distributed noise. The SNR was then computed as

$$\text{SNR} = 20 \log_{10}\left(\frac{A_{\text{p-p}}}{\sigma_{\text{noise}}}\right),$$

with $A_{\text{p-p}}$ denoting the peak-to-peak signal amplitude (in mAU). Using this approach, the global SNR was approximately 66 dB, while local SNR values ranged from 31 to 40 dB depending on window size ($w = 50 \ldots 500$ points). Estimates stabilized for $w \geq 250$, corresponding to a noise level of $\approx$ 0.02 mAU and a signal amplitude of $\approx$ 37 mAU. Figures S1.1 and S1.2 summarize the dataset-level distributions used for simulator calibration and statistical characterization. These distributions informed the calibrated ranges for duration and area simultaneously, ensuring the synthetic generator covers both the most common and the extreme peak types.

Text S1.2. Data cleaning and filtering criteria

The primary data-cleaning steps involved the removal of duplicate and incomplete entries. For the statistical analysis of real SEC traces and for calibrating the synthetic data generator, additional filtering was applied to exclude injections not representative of routine product analytics (e.g., maintenance or cleaning runs). Filtering criteria were defined together with subject matter experts (SMEs) and covered five categories:

- Validity of sample metadata, including Uniform Resource Locator (URL) and sample name consistency.
- Correct detector channel, ensuring that traces corresponded to the default analytical channel within the parent sequence.

- Peak-count plausibility, retaining only chromatograms with ≤ 30 peaks, consistent with SEC expectations for therapeutic proteins.

- Validity of peak features, requiring nonzero peak area and excluding non-quantitative peak types such as "riders."

- Appropriate chromatography method, retaining only SEC, preparative SEC (prepSEC), or quantitative SEC (qSEC) methods.

These filters ensured that only analytically meaningful, method-relevant chromatograms were used for statistical characterization and simulator calibration. Figure S1.1 summarizes peak area proportions, peak durations, and sequence lengths before and after resampling, while Figure S1.2 summarizes the temporal distributions of peak events together with the corresponding signal-to-baseline differences at those event positions. Together they capture the analyst-driven event conventions that guided the labeling and the simulator's windowing policy.

For real-data benchmarking, we further curated a high-consensus subset of 412 chromatograms spanning ten large-molecule development projects. The set is dominated by antibodies and antibody-derived formats, reflecting the intended application domain of the study while preserving diversity in molecular architecture and molecular weight. Table S1.1 summarizes this project-level composition.

Project	Chromatogram count	Format type	Mol. weight (kDa)
A	56	DutaMab	145
B	61	1+1 CrossMab	145
C	2	2+1 CrossMab	195
D	86	MAb with C-terminal peptide fusion	159
E	45	MAb with C-terminal CrossFab fusion	191
F	9	3+1 CrossMab	239
G	95	1+1 CrossMab	145
H	6	Fc fusion protein	143
I	16	1+1 CrossMab	144
J	36	1+1 CrossMab with N-terminal cytokine fusion	164

Table S1.1: Project-level composition of the curated GENA real-data benchmark used for evaluation. Counts sum to 412 chromatograms across ten antibody and antibody-derived large-molecule programs.

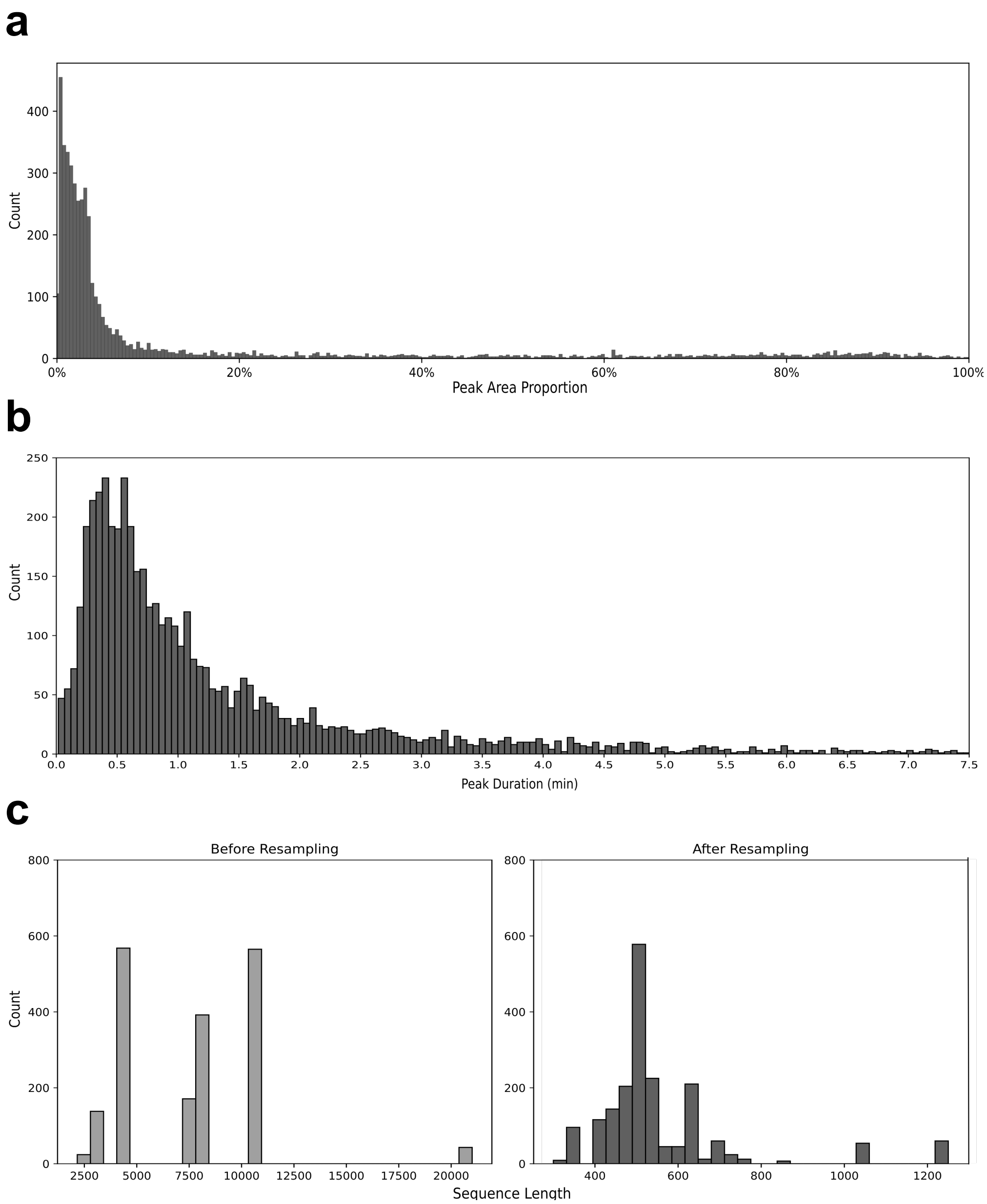

Figure S1.1: Dataset-level distributions used for simulator calibration and benchmark characterization. (a) Peak area proportions as percentage of total chromatogram area. Most peaks contribute less than 5% of the total signal, with smaller clusters corresponding to dominant product peaks. (b) Peak-duration distribution. The distribution is right-skewed, with a primary concentration between 0.3 and 0.7 min and a long tail toward broader peaks. (c) Sequence-length distribution before and after resampling. Distinct raw acquisition-length clusters collapse into a narrower standardized range after resampling. (Sample of 5000 chromatograms) Created in BioRender. Walter, D. (2026) https://BioRender.com/w3s3dyc.

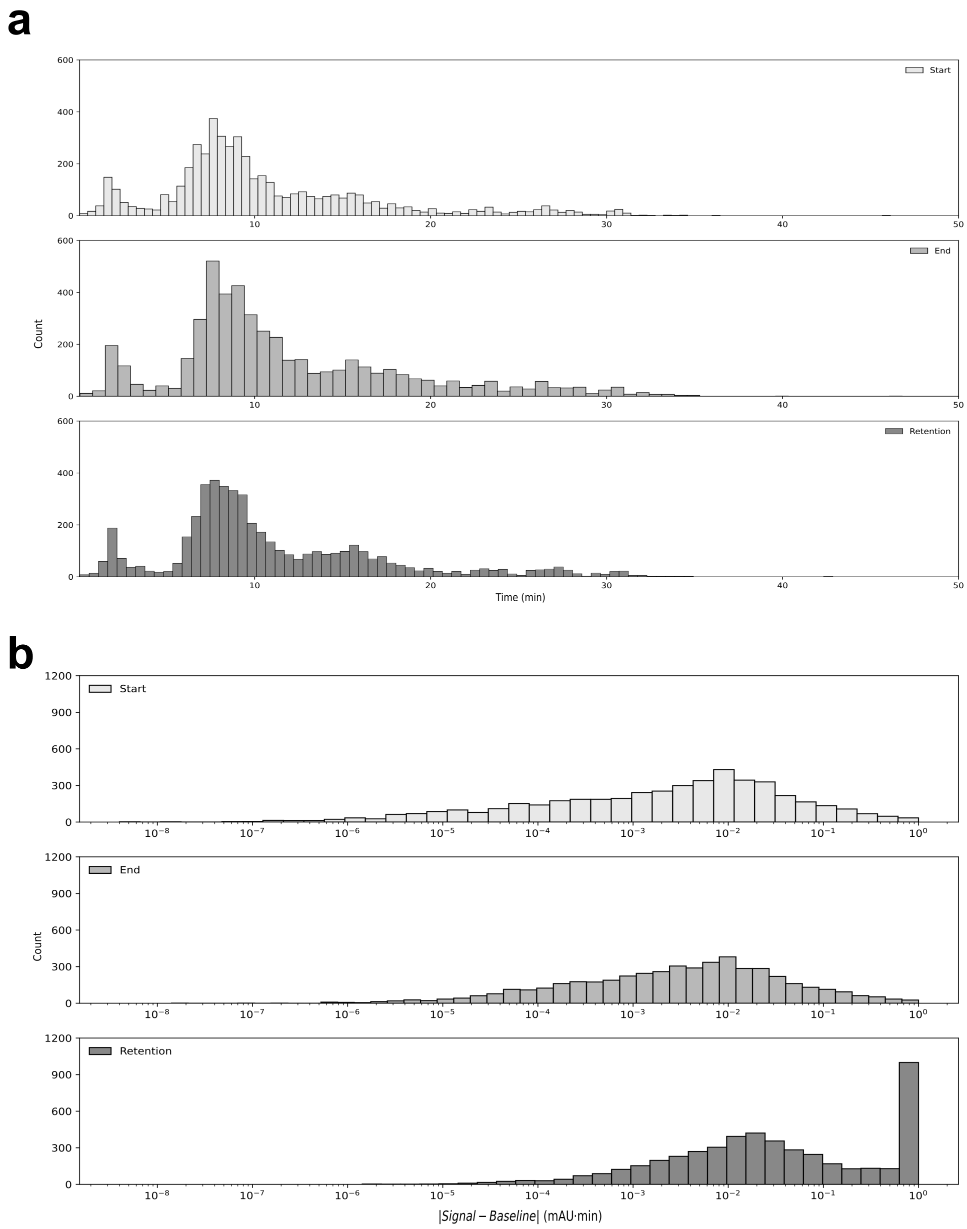

Figure S1.2: Dataset-level distributions of peak-event timing and event-associated signal amplitudes. (a) Temporal distributions of peak start, end, and retention events. The highest concentration occurs around 9 min, with a smaller early cluster around 3 min. (b) Distributions of |signal − baseline| at the corresponding event positions on a logarithmic scale, showing distinct amplitude regimes for boundary and retention events. (Sample of 5000 chromatograms) Created in BioRender. Walter, D. (2026) https://BioRender.com/qo2mtsd.

Text S1.3. Empirical comparison of synthetic and real windows

Figure S1.3 compares six window-level summary statistics across synthetic data, curated real GENA windows, and a separate real SEC sample. Panels a and b summarize signal characteristics. The synthetic data cover the signal-to-noise and signal-concentration regimes observed in both real datasets, although the signal-to-noise distribution is somewhat broader. Panels c and d summarize peak characteristics. Peak duration is similar between the synthetic and GENA data, whereas the separate SEC sample more often contains peaks occupying a smaller fraction of the window. For peak area share, the synthetic distribution is more discrete, reflecting the more regular relative peak-area structure of the simulated windows. Panels e and f summarize label characteristics. Peak-labeled fraction is similar between the synthetic and GENA data, whereas the separate SEC sample contains smaller labeled fractions per window. Label fragmentation is higher in the separate SEC sample than in the synthetic and GENA data, indicating a domain shift in label structure rather than a simple difference in difficulty.

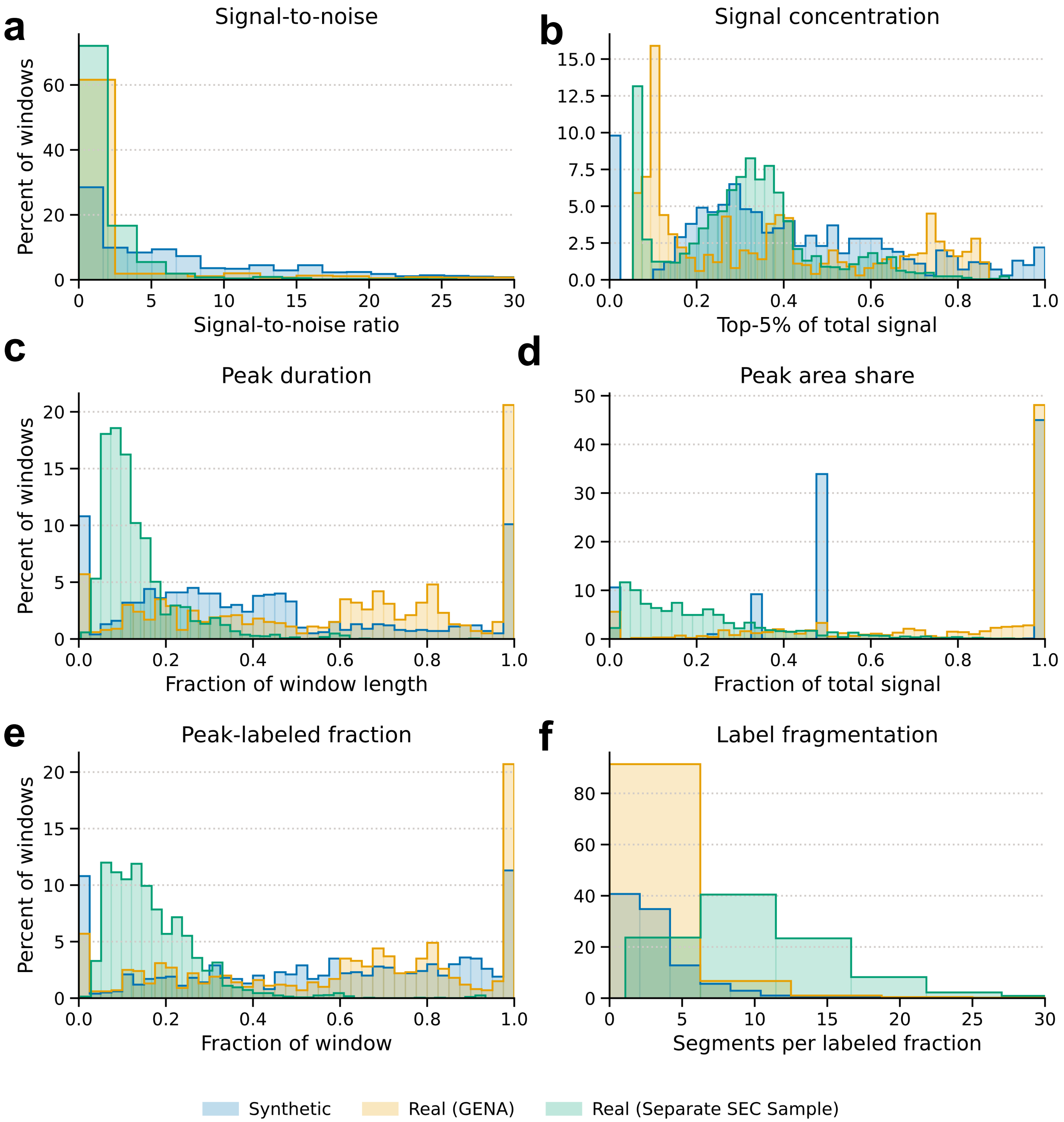

Figure S1.3: Window-level comparison of synthetic and real SEC signal characteristics. The six panels summarize signal-to-noise, signal concentration, peak duration, peak area share, peak-labeled fraction, and label fragmentation for synthetic windows, curated real GENA windows, and a separate real SEC sample from the broader routine corpus. The comparison highlights a structured domain shift: GENA windows tend to exhibit higher signal-to-noise ratios and broader, more dominant peaks than synthetic windows, whereas the separate SEC sample is substantially noisier and more fragmented. Created in BioRender. Walter, D. (2026) https://BioRender.com/9vohc6w.

S2 Simulator calibration

Text S2.1. Peak parameterisation and peak-type variants

Peak locations, amplitudes, and widths in the simulator were constrained to empirically calibrated ranges derived from the quality-controlled chromatograms. Peaks were categorised by amplitude (very high, high, medium, low, very low) and by width (narrow, wide). Narrow peaks correspond to standard deviations between 0.005 and 0.03 (approximately 5–30 s), wide peaks to 0.02–0.09 (approximately 20–90 s). Very low peaks extend down to an amplitude of $1{\times}10^{-4}$ to allow realistic low-level and baseline-adjacent signals.

Peak centres were sampled from three partially overlapping retention regions ("left", "middle", "right") and a global region for medium and low peaks, which biases very high and very low peaks toward early and late elution while keeping medium and low peaks concentrated in a broader central-to-late elution region. A minimal centre-to-centre separation of 0.3–0.5 units was enforced between neighbouring peaks to avoid unrealistic overlap. Table S2.1 summarises the peak categories and their parameter ranges, and the retention windows can be expanded to left = [-0.5, 0.4], middle = [0.3, 0.7], right = [0.6, 1.5], global = [0.7, 1.0]; Figure S2.1 illustrates how split-normal parameter variations modulate height, centre, and asymmetry across these morphologies.

Text S2.2. Peak-type probabilities and variants

Base occurrence probabilities for each peak type followed a heavy-tailed profile with rare very high peaks and frequent low and very low peaks (e.g., base probabilities of 0.01–0.02 for very high and high types, 0.05–0.1 for medium and low types, and 0.1 for very low types). On top of this base distribution, several peak-pattern variants were defined to induce structured diversity:

- Positional variants ("left", "middle", "right"): restrict peaks to early, central, or late retention regions, respectively. Each positional variant is sampled with probability 0.1 and zeros the probabilities of peaks outside the selected region.
- Large/medium variant: sampled with probability 0.05, increases the probabilities of very high, high, and medium peaks while suppressing low and very low peaks.
- Small-peak variant: sampled with probability 0.2, increases the probabilities of low and very low peaks and suppresses high and very high peaks.
- Mixed small-and-large variant: sampled with probability 0.6, retains the base heavy-tailed structure and co-occurrence of very low and very high peaks.

Variants are realised through multiplicative profiles and overrides applied to the base peak-type distribution, which allows the simulator to generate traces with few large peaks, many small peaks, or localized clusters while staying consistent with the calibrated parameter ranges.

Signals were perturbed in two stages to emulate instrument variability: a short-term noise process sampled from white, gaussian, uniform, and green noise families and a low-order baseline component implemented as a constant offset or shallow quadratic drift. Figure S2.2 shows the canonical noise profiles at three representative amplitude levels, illustrating how each spectral family contributes distinctive variability before the random cropping and window generation steps.

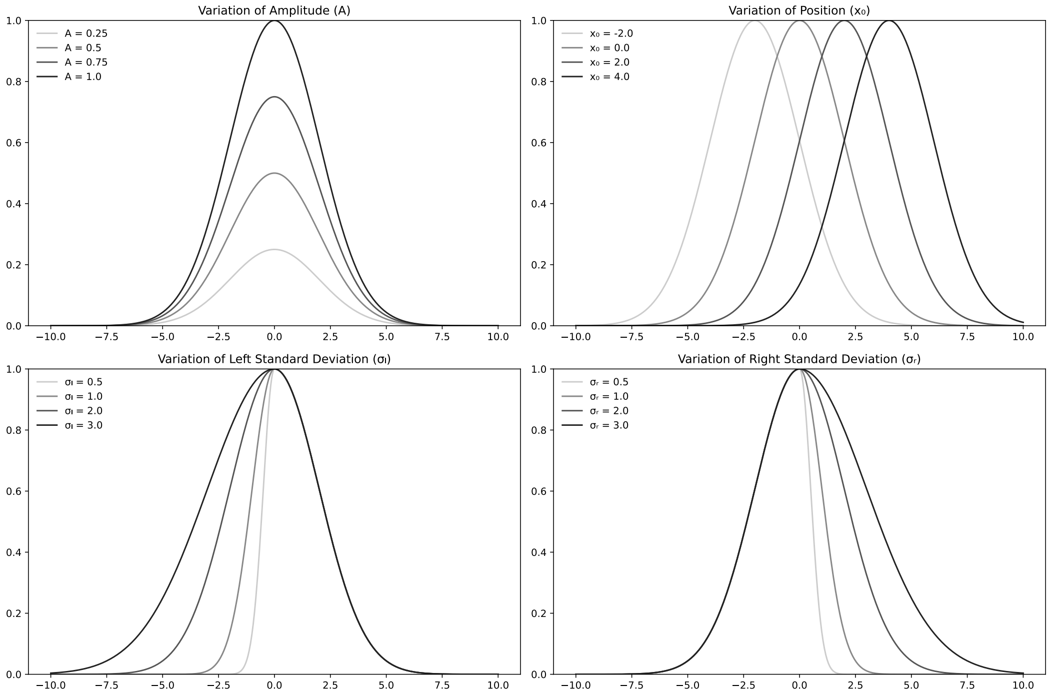

Figure S2.1: Parameter variations in the split-normal distribution, showing effects on peak morphology. Top-left: amplitude (A) variations affect peak height. Top-right: position (x_0) shifts move the peak horizontally. Bottom-left/right: left (σ_L) and right (σ_R) standard deviation changes control fronting and tailing, respectively.

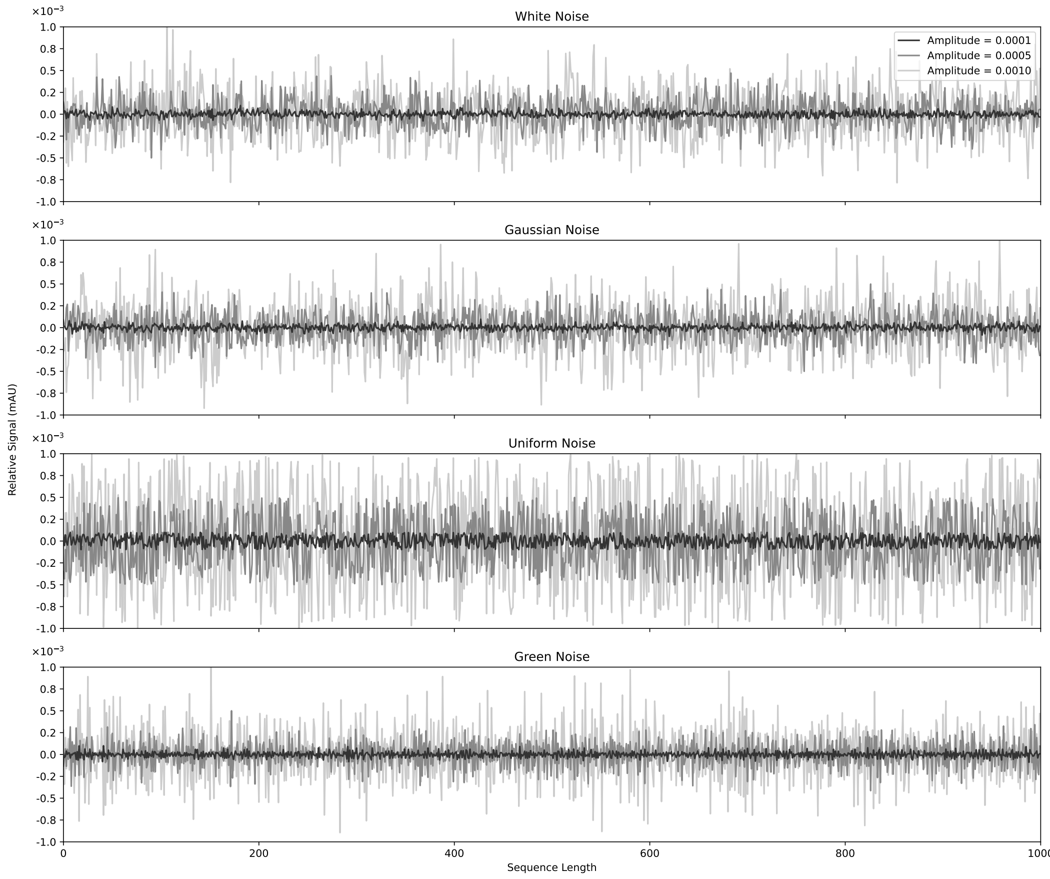

Figure S2.2: Noise profiles used in the simulator, shown at three amplitude levels (0.0001, 0.0005, 0.0010) for each family: white, gaussian, uniform, and green noise.

Category	Width	Centre window	Amplitude range	Std range
Very high	Narrow	left/middle/right	0.6–1.0	0.005–0.03
Very high	Wide	left/middle/right	0.6–1.0	0.02–0.09
High	Narrow	left/middle/right	0.3–0.6	0.005–0.03
High	Wide	left/middle/right	0.3–0.6	0.02–0.09
Medium	Narrow	global	0.1–0.3	0.005–0.03
Medium	Wide	global	0.1–0.3	0.02–0.09
Low	Narrow	global	0.05–0.1	0.005–0.03
Low	Wide	global	0.05–0.1	0.02–0.09
Very low	Narrow	left/middle/right	$1{\times}10^{-4}$–0.05	0.005–0.03
Very low	Wide	left/middle/right	$1{\times}10^{-4}$–0.05	0.02–0.09

Table S2.1: Peak parameter ranges used for simulator calibration. Amplitude classes are paired with width categories; centres are sampled from the indicated retention windows. Minimal distance between peak centres: 0.3–0.5 units.

S3 Peak shape and chromatogram generation

Text S3.1. Split-normal peak model

Individual peaks were modelled using a split normal (two-piece normal) functional form with a common centre μ, amplitude A, and side-specific standard deviations σ_L and σ_R. The intensity at position x reads

$$p(x) = \begin{cases} A \exp\left[-\frac{1}{2}\left(\frac{x-\mu}{\sigma_\mathrm{L}}\right)^2\right], & x < \mu, \\ A \exp\left[-\frac{1}{2}\left(\frac{x-\mu}{\sigma_\mathrm{R}}\right)^2\right], & x \geq \mu. \end{cases}$$

This amplitude-scaled formulation was not normalised as a probability density.

Text S3.2. Parameter sampling and asymmetry realisation

For each chromatogram, the number of peaks was sampled uniformly from $[1, N_\mathrm{max}]$. Each peak category was drawn from the variant-adjusted probability distribution, after which centre μ, amplitude A, and base width σ were sampled from the calibrated ranges. Asymmetry was introduced by sampling independent factors f_L and f_R and converting them to side-specific widths,

$$\sigma_\mathrm{L} = \sigma(1 - f_\mathrm{L}), \qquad \sigma_\mathrm{R} = \sigma(1 + f_\mathrm{R}),$$

yielding sharper rise and broader tails depending on the sampled factors.

Text S3.3. Peak extent, overlap control, and boundary adjustment

Start and end boundaries were defined by positions where the intensity decayed to $A\alpha$ with $\alpha = 10^{-5}$; solving the split-normal yields

$$x_\mathrm{start} = \mu - \sigma_\mathrm{L}\sqrt{-2\ln(\alpha)}, \qquad x_\mathrm{end} = \mu + \sigma_\mathrm{R}\sqrt{-2\ln(\alpha)}.$$

Candidate peaks were sorted by centre position and filtered if they violated a minimum centre-to-centre distance (which scales with local width) or if their cutoff-defined extents overlapped excessively. Residual overlap was resolved by numerically computing the intersection of adjacent split-normal curves within $[\mu_1, \mu_2]$ and updating the corresponding boundaries.

Text S3.4. Chromatogram synthesis, normalisation, and event labels

The final chromatogram sums all individual peak functions. When the maximum intensity exceeded unity, the entire trace and its component peaks were rescaled by the same factor to keep signals within $[0, 1]$ while preserving relative heights. Peak-count labels count how many peaks exceed the cutoff per position; start/end events follow label transitions, retention indices coincide with μ, and shoulders are recorded where adjacent peaks share a split-point intersection.

S4 Model configuration, training, and hardware

Text S4.1. Default model card

The reported Peak Feature Extractor 1 (PFE-1) configuration employs eight encoder layers with eight attention heads, an embedding width of 256, and a feed-forward dimension of 2048. Scalar inputs are projected through learnable linear layers, concatenated with index-based encodings and Time2Vec (T2V) components (kernel size 31), and processed by GELU-activated transformer blocks. Attention paths alone receive dropout (0.2), while residual projections and readout heads remain deterministic to preserve calibration. Region outputs carry class weights (2:1:2) for baseline/peak/collision, and event heads use $(50, 80, 80, 50)$ to emphasize retention/end decisions. Three million simulator windows (shifted regime) are partitioned into train/validation/test splits using an 80/10/10 ratio, yielding 2.4×10^6 training windows per epoch. Gradient accumulation and parallelization yield an effective global batch size of 496 windows, enabling AdamW (learning rate 1×10^{-4}, weight decay 0.01, step size ten epochs, $\gamma = 0.05$) to train for up to twenty epochs with early-stopping patience of two epochs; best validation checkpoints were typically reached around epoch fifteen. Runtime diagnostics and resource footprint are summarized in Table S4.1.

Text S4.2. Training pipeline and runtime diagnostics

Runtime logs capture the effective footprint of the above configuration. Twenty epochs process 4.8×10^7 training windows (2.4×10^6 per epoch), consuming roughly 1.29×10^5 s (≈ 36 h) wall clock with a median epoch duration of 6469 s. Throughput on the production pass averaged 3.86×10^2 windows s^{-1}, and peak VRAM allocation stayed below 17.3 GB despite its tens-of-millions-scale parameter count. Gradient norms peaked in the low-thousands during burn-in and decayed to single digits near convergence, mirroring the plateau behavior seen in the fixed-grid tuning summary (Text S5.2). Validation checkpoints occurred every 300 000 windows (605 mini-batches), matching the cadence used for downstream evaluation. Deterministic seeds, frozen cuDNN kernels, and scripted dataloader permutations were enforced throughout; aggregate seed statistics are provided in Text S5.3. Table S4.1 condenses the runtime diagnostics retained in the internal logs.

Text S4.3. Compute environment

Experiments ran on Roche pRED's Kaiseraugst (Switzerland) HPC complex. Production jobs used HPE Apollo GPU nodes equipped with dual AMD EPYC processors (≈ 32 cores each), several hundred gigabytes of RAM, and four NVIDIA A100 GPUs (40 GB) per node;

smaller supporting runs sometimes used V100 32 GB or L40S 48 GB accelerators. CPU preprocessing relied on dual Intel Xeon Platinum nodes (≈ 48 cores). All nodes share NVMe M.2 scratch storage and IBM ESS-backed filesystems, interconnected via Mellanox HDR100 (100 Gbit s^{-1}). Job dispatch is handled by LSF/SLURM, providing reproducible scheduling for the 36 h training runs described here.

Metric	Value
Trainable parameters	$\sim 2.8 \times 10^7$
Peak VRAM allocation	17.3 GB (four NVIDIA A100 40 GB)
Effective batch size	496 windows (global, per-step)
Samples processed	4.8×10^7 windows over 20 epochs
Median epoch duration	6469 s (median), 6469 s (mean)
Total wall-clock runtime	1.29×10^5 s ($\approx$ 35.9 h)
Throughput	3.86×10^2 windows s^{-1}
Peak gradient norm	1.5×10^3 (epoch 1), decaying to < 10 by epoch 10
Validation cadence	300 000 windows (605 batches) per checkpoint
Deterministic controls	Fixed seeds, cuDNN determinism, scripted data order

Table S4.1: Runtime diagnostics and resource footprint for the PFE-1 training run underlying the manuscript results.

S5 Hyperparameter sweeps, diagnostics, and reproducibility

Text S5.1. Sobol grids and contour plots

Sobol-sampled grids explored learning rate, weight decay, dataset size, and representation width simultaneously. Contour plots identify a narrow corridor of stable convergence: Figure S5.1 contrasts learning rate and weight decay for varying dataset sizes, Figure S5.2 highlights the same corridor in embedding versus hidden dimensions, and Figure S5.3 maps encoder depth versus attention heads at three million chromatograms. These contours guided the final configuration and underpin the parameter ranges referenced in the main text.

Text S5.2. Fixed grids: depth, heads, and scale

Fixed-grid sweeps complement the contour views above. Figure S5.4 summarizes head-count, dataset-size, and encoder-depth scans in a common format. The dot-connected traces illustrate that performance gains plateau beyond the selected configuration, while larger variants increase variance and training time. These sweeps corroborate the narrow operating band cited in the main text.

Text S5.3. Seed variance and reproducibility diagnostics

Twenty independent seeds retrained the published architecture on the same synthetic corpus. Event-F_1 exhibits the largest seed sensitivity (mean 0.381, standard deviation 0.066, 95 % CI [0.353, 0.410]), whereas region-F_1 and normalized Box-Loss remain tightly clustered (means 0.980, standard deviations 0.009 and 0.005; 95 % CIs [0.976, 0.983] and [0.977, 0.982], respectively).

Text S5.4. Box-loss weight balancing

The normalized, intensity-weighted Box-Loss combines per-box intensity shares $w(B)$ with class-specific scalars $\lambda_{\mathrm{TP}}, \lambda_{\mathrm{FN}}, \lambda_{\mathrm{FP}}$. IoU is computed on signal-derived rectangles obtained from a temporal interval and its local signal range, as described in the Methods. In our implementation, $w(B)$ is computed by summing the per-timepoint intensity-share signal over the temporal support of a box, with optional sub-linear scaling and minimum-penalty handling for edge cases; false-positive contributions may be damped to reduce sensitivity to spurious intervals in high-signal regions. By default the solver uses $\lambda_{\mathrm{TP}} = 1.0$, $\lambda_{\mathrm{FN}} = 8.0$, and $\lambda_{\mathrm{FP}} = 2.0$, emphasizing the penalty for missed peaks while keeping true positives sensitive to IoU deviations.

For each chromatogram n, the raw mismatch is

$$P_{\text{box}}^{(n)} = \lambda_{\text{TP}} P_{\text{TP}}^{(n)} + \lambda_{\text{FN}} P_{\text{FN}}^{(n)} + \lambda_{\text{FP}} P_{\text{FP}}^{(n)}.$$

Dataset totals are obtained by summation over D: $P_{\text{box}}(D) = \sum_{n \in D} P_{\text{box}}^{(n)}$. To define the normalization anchor, we evaluate the same loss terms on a fixed reference prediction $\mathcal{B}_{\text{full}}^{(n)} = \{(0, T_n)\}$, where T_n denotes the last valid time index of chromatogram n. This yields the dataset-specific reference penalty $P_{\text{max}}(D)$. The reported score is

$$L_{\text{box,norm}}(D) = \frac{P_{\text{max}}(D) - P_{\text{box}}(D)}{P_{\text{max}}(D)},$$

so values closer to 1 indicate better agreement. When component-level reporting is required, TP/FN/FP components are normalized analogously against their corresponding reference totals $P_{\text{TP,max}}(D)$, $P_{\text{FN,max}}(D)$, and $P_{\text{FP,max}}(D)$, each computed from the same full-span reference construction. These scalars and normalization references can be tuned to align with different tolerance trade-offs.

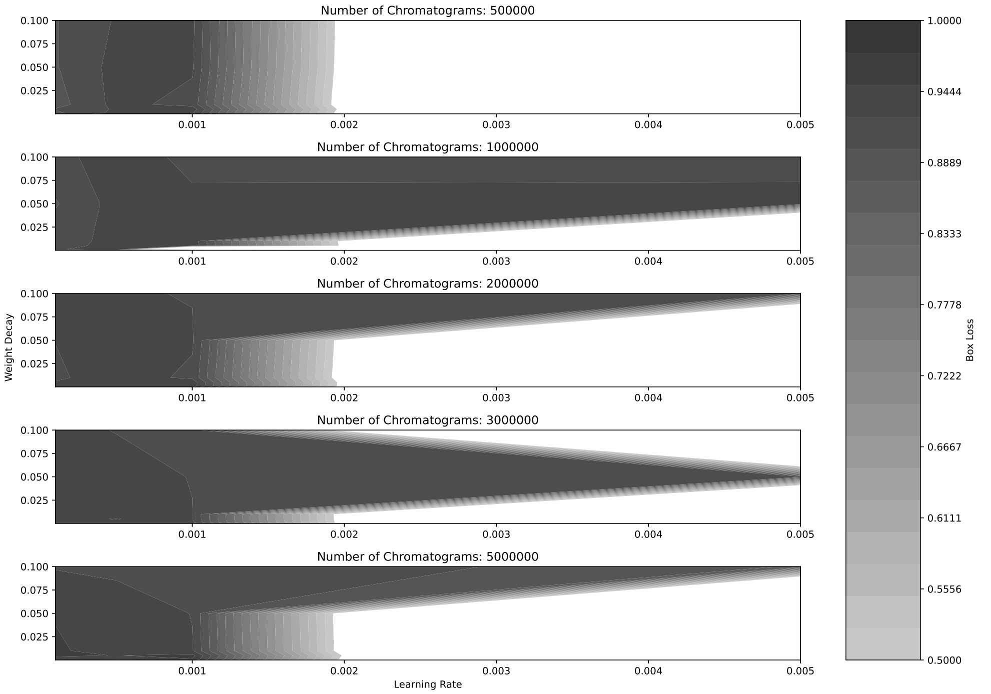

Figure S5.1: Contour maps of validation Box-Loss across learning rate (x-axis) and weight decay (y-axis) for multiple dataset sizes (500k, 1.5M, 3M). The highlighted corridors trace where convergence stability and generalization align best.

Text S5.5. Robustness of Box-Loss to matching and weighting variants

We tested whether the model ranking depends strongly on one specific choice of matching rule, overlap metric, or TP/FN/FP weighting. Six plausible variants were evaluated by combining greedy or Hungarian matching, rectangle-IoU or segment-based overlap measures, and three nearby weighting schemes. Across all tested variants, the ranking remained unchanged on both datasets: PFE-1 scored highest, followed by the convolutional neural network (CNN) baseline and then Savitzky–Golay (SG). On synthetic data, the explored ranges remained ordered throughout (PFE-1: 0.943–0.968; CNN: 0.763–0.902; SG: 0.340–0.510). On GENA, the ranges were narrower overall and again ranking-stable (PFE-1: 0.865–0.929; CNN: 0.811–0.894; SG: 0.827–0.873). These results indicate that the main benchmark conclusion is not specific to one Box-Loss variant.

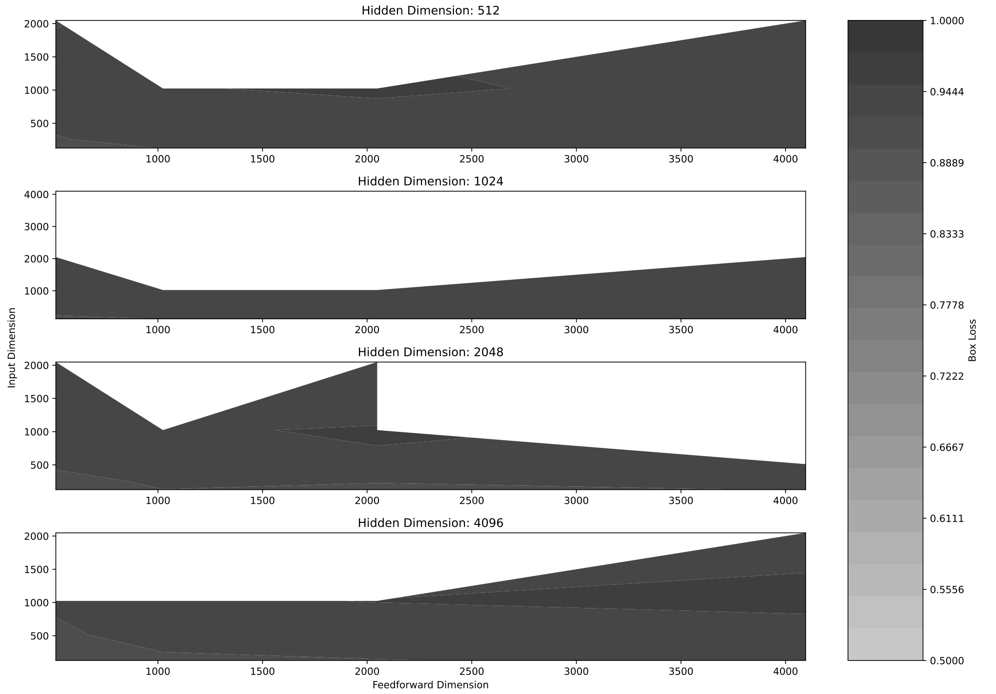

Figure S5.2: Contour plots relating input/embedding width and feed-forward projection width across different depth configurations. Optimal regions tighten as the architectural depth increases, justifying the selected 256/2048 combination.

Text S5.6. Component-wise interpretation of Box-Loss

To clarify which error types drive the aggregate score, we decomposed the reference Box-Loss into TP, FN, and FP contributions. On synthetic data, SG is strongly false-negative driven, indicating that it misses many weaker or interacting peaks. The CNN baseline reduces false negatives substantially but incurs a larger false-positive penalty. PFE-1 achieves the lowest total loss by lowering TP mismatch, FN penalties, and FP penalties simultaneously. On GENA, the same logic holds in attenuated form: SG remains conservative and FN-heavy, CNN is more balanced, and PFE-1 achieves the lowest total loss. This decomposition explains why the best Box-Loss model is not necessarily the most conservative one.

Text S5.7. Boundary tolerance as a complementary view

As a complementary analysis, we evaluated start/end boundary detection independently of the box metric by matching predicted boundaries within tolerance windows of 2, 5, or 10 indices. On synthetic data, PFE-1 remains superior under all tolerances. On GENA, the difference between PFE-1 and the CNN baseline is smaller under the strictest tolerance, but PFE-1 separates more clearly as the tolerance window relaxes. Boundary tolerance and

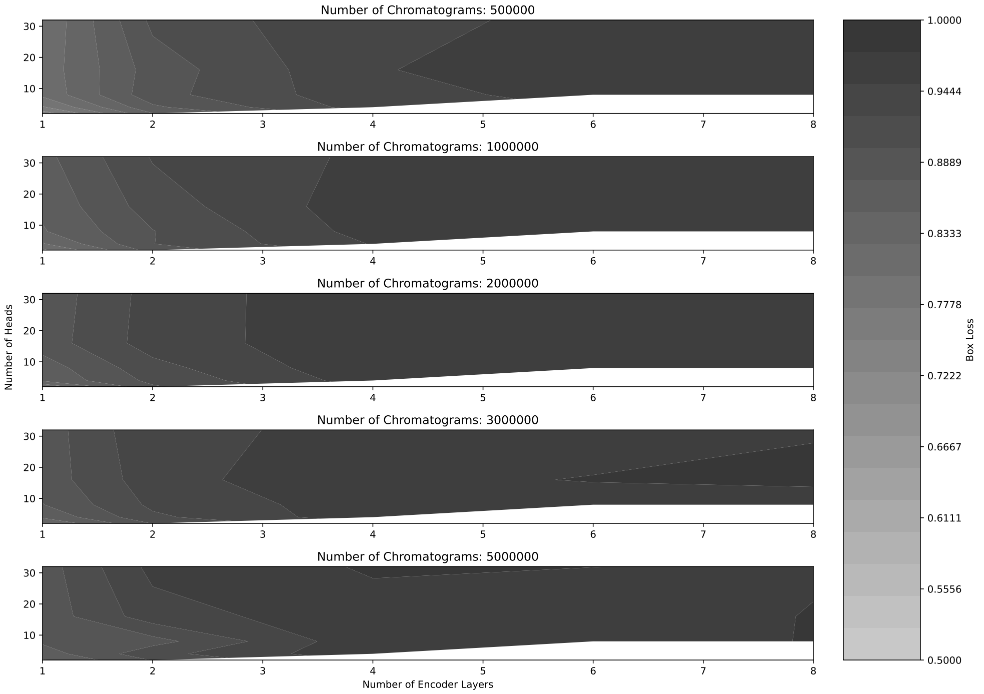

Figure S5.3: Contour plot of encoder depth (x-axis) versus attention heads (y-axis) for models trained on 3 million chromatograms. The L-shaped optimum spans roughly 4–8 layers and 12–24 heads; white areas denote invalid configurations where the pairings were not evaluated.

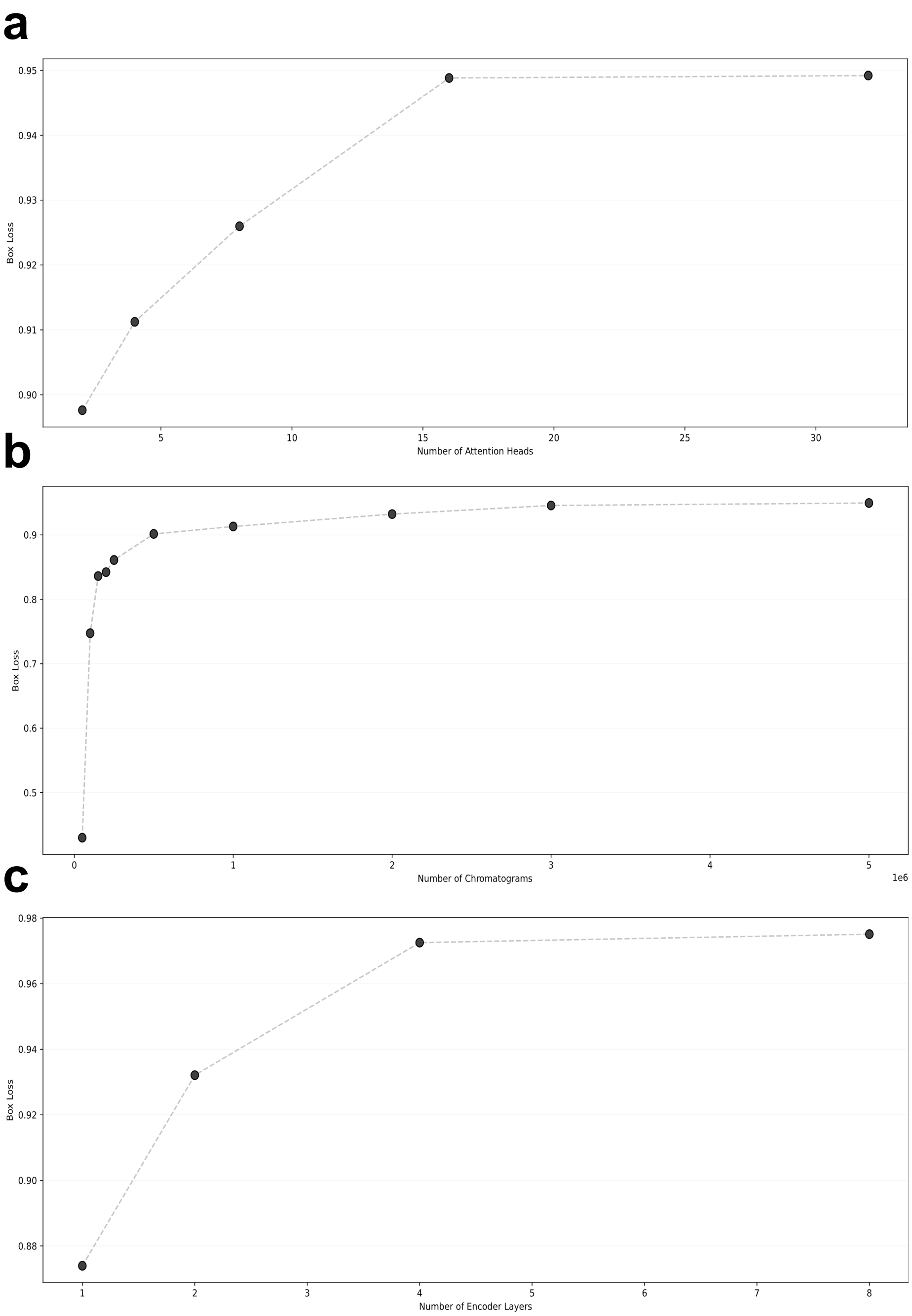

Figure S5.4: Fixed-grid sweeps for (a) number of attention heads vs Box-Loss, (b) number of chromatograms vs Box-Loss, and (c) number of encoder layers vs Box-Loss. The connected traces show that performance improves rapidly from small configurations and then flattens within the selected operating corridor. Created in BioRender. Walter, D. (2026) https://BioRender.com/267zgr8.

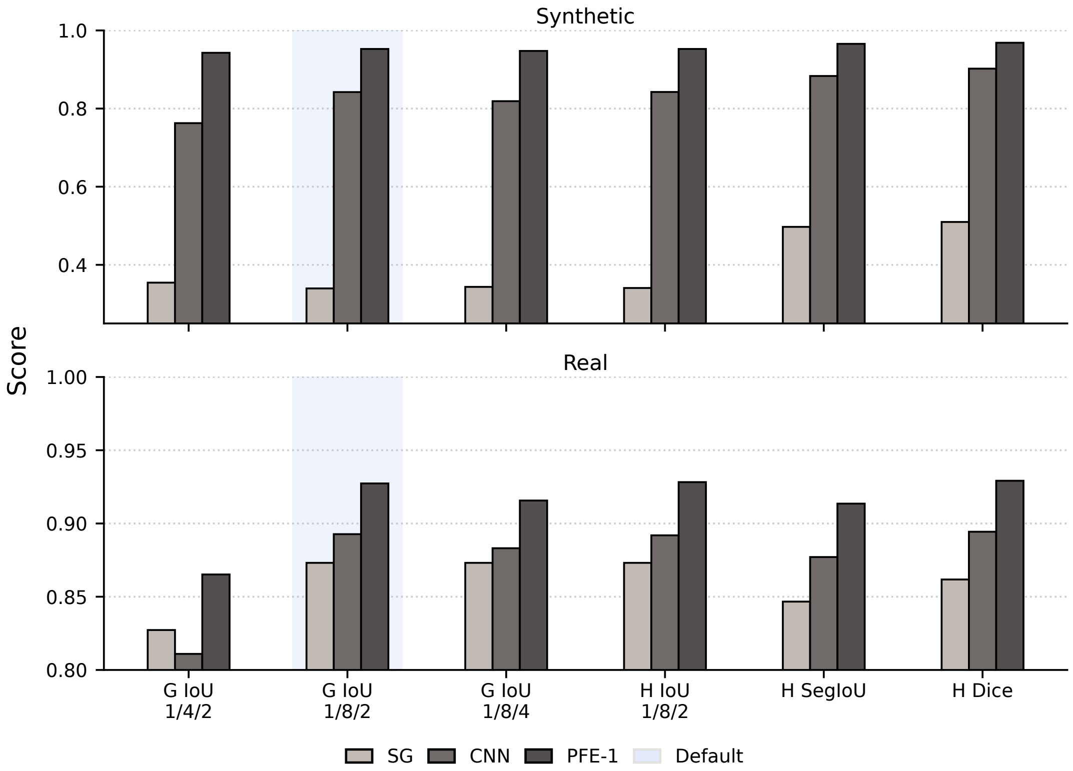

Figure S5.5: Box-Loss robustness under six plausible matching and weighting variants on synthetic and GENA data. Absolute scores shift across variants and datasets, but the overall ranking PFE-1 > CNN > SG is preserved. The lightly shaded blue region marks the reference configuration used in the main evaluation.

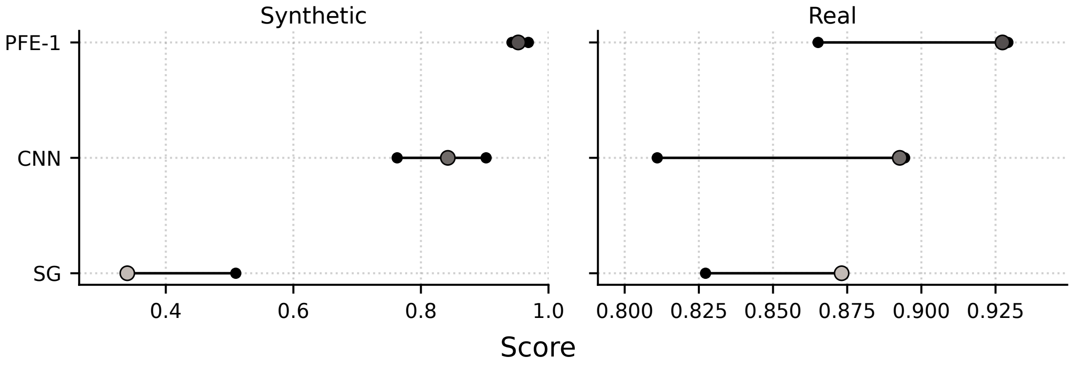

Figure S5.6: Model stability across the tested Box-Loss variants on synthetic and GENA data. Black endpoints show the minimum and maximum score reached by each model, and the colored marker indicates the reference Greedy IoU 1/8/2 configuration.

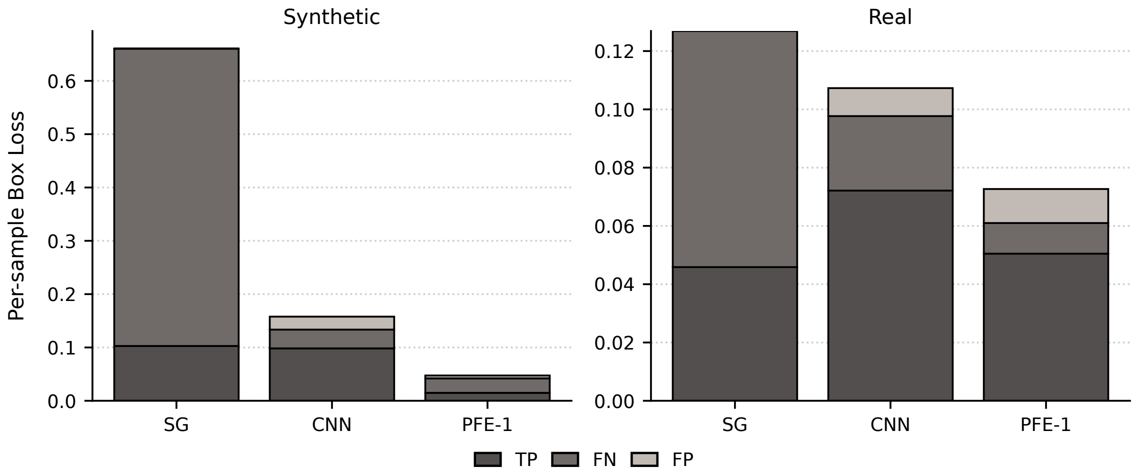

Figure S5.7: Per-sample decomposition of the reference Box-Loss into TP, FN, and FP contributions on synthetic and GENA data. Lower total stacked height indicates better agreement. The derivative baseline is dominated by missed peaks, whereas PFE-1 achieves the lowest total mismatch on both datasets.

Box-Loss therefore capture related but not identical aspects of prediction quality.

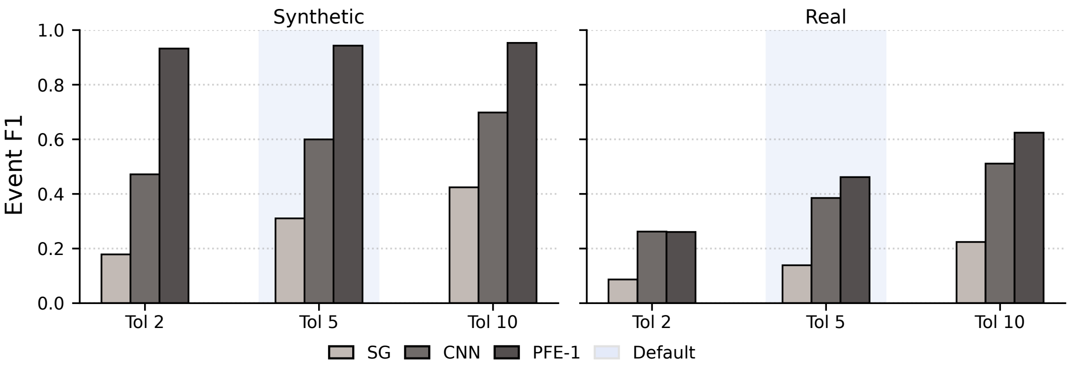

Figure S5.8: Boundary-tolerance analysis for start/end events on synthetic and GENA data. Under the strictest real-data tolerance, PFE-1 and the CNN baseline are similar, whereas PFE-1 separates more clearly as the tolerance window widens.

Text S5.8. Sensitivity to solver rules

To separate model behavior from deterministic post-processing, we varied simple solver rules after box generation and measured score changes relative to the dataset-specific reference setting. These rules are intended to correct recurrent artifacts in the real-data transfer setting rather than to redefine the prediction task. One example is local shoulder suppression when shoulder and retention events are co-activated in a pattern that would otherwise split a main peak.

Changing individual solver rules perturbs these scores, but most settings remain near the reference configuration. On synthetic data, nearby settings such as minimum box length 1 or 5 slightly improve the score (0.973 and 0.973 versus 0.953), whereas stronger interventions such as minimum box length 20 or disabling the shoulder-drop rule degrade performance more clearly (0.826 and 0.857, respectively). On GENA, most tested settings cluster around 0.933, with the strongest degradation observed for minimum box length 20 (0.924) and modest improvements for higher peak-height or prominence thresholds (up to 0.934). These sweeps show that the solver affects the final score, but also that improvements on real data require relatively conservative adjustments.

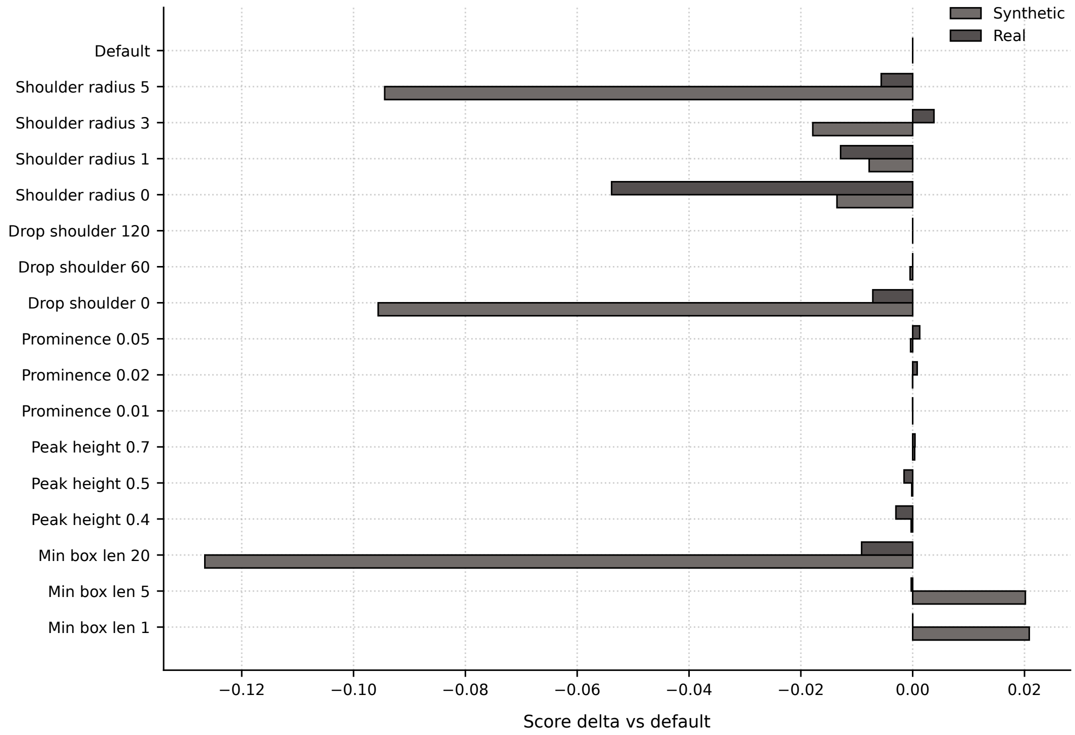

Figure S5.9: Sensitivity of the final box score to solver-rule changes, expressed as deviations from the dataset-specific reference setting on synthetic and GENA data. Most settings remain close to the reference configuration, indicating moderate rather than extreme solver fragility. The lightly shaded blue region marks the reference setting used in the main evaluation.

Text S5.9. Sensitivity to stride and windowed reconstruction

Because full chromatograms are processed by overlapping fixed-length windows, the final output additionally depends on how window predictions are merged along the time axis. We therefore evaluated uniform, Hann, and Tukey windowing across GENA stride values from 1 to 250. The resulting curves show a pronounced non-monotonic dependence on

reconstruction settings: very small strides produce relatively high merged region-F_1 values near 0.52 but much lower final box scores around 0.82, whereas intermediate strides yield the strongest box-level agreement. This divergence indicates that dense overlap can smooth local region predictions without necessarily improving the final peak intervals recovered after solver post-processing.

The window-function choice matters less than the stride itself. Around the strongest-performing stride range, uniform, Hann, and Tukey merging are very similar on the final score. The U-shaped trend is more pronounced for merged region-F_1 than for Box-Loss, which follows a broader inverse-U pattern. Window functions therefore primarily affect reconstruction into final boxes rather than the underlying local predictions.

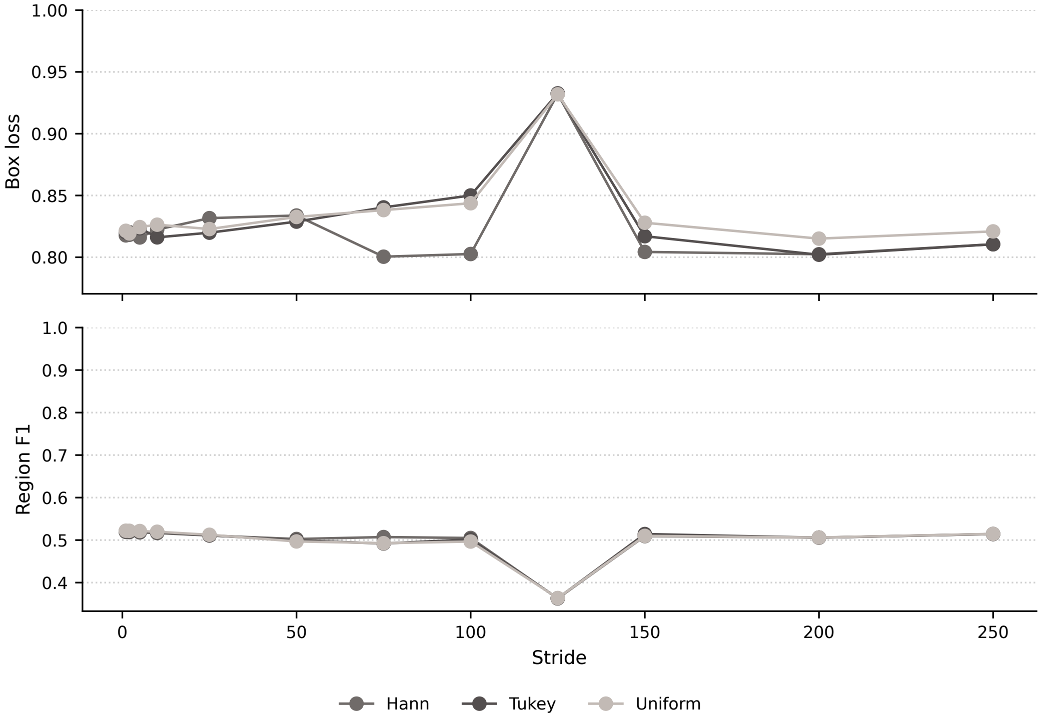

Figure S5.10: GENA sensitivity to reconstruction stride and window function. The upper panel shows the final Box-Loss and the lower panel the merged region-F_1; small strides can improve merged region predictions without improving the final peak boxes recovered after solver-based reconstruction.

Text S5.10. Capacity analysis within the PFE family

To contextualize the selected architecture within the broader PFE family, we evaluated a dense sweep over encoder depth while keeping the remaining configuration fixed. The validation-loss sweep shows that optimization improves rapidly from shallow models to the

mid-depth range and then approaches a plateau from roughly 6 to 8 layers onward. The variance is largest at 5 layers, whereas deeper configurations remain comparatively stable within the observed range.

The corresponding Box-Loss sweep completes the same picture at evaluation time. On synthetic data, performance exceeds both baselines from 2 layers onward and then remains in a narrow high-performing band from roughly 3 to 8 layers, with the strongest results at 7 to 9 layers. On GENA, all tested configurations outperform the baselines, and larger models mainly reduce seed variance rather than producing a single sharp optimum. We therefore treat this dense sweep as supportive evidence: the selected configuration lies within a stable high-performing regime, while smaller configurations may remain sufficient for settings where a smaller parameter budget is advantageous.

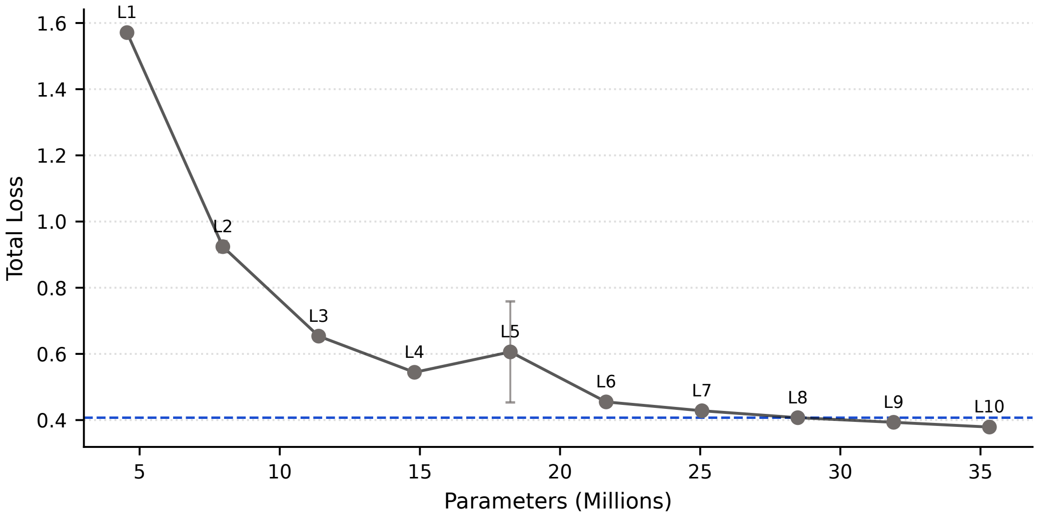

Figure S5.11: Best validation total loss across a dense encoder-depth sweep within the PFE family. Optimization improves rapidly from shallow to mid-depth models and then approaches a plateau. The horizontal blue line marks the selected configuration used in the present study. Error bars indicate seed-to-seed variation where visible; points without visible error bars have near-zero deviation at the plotted scale.

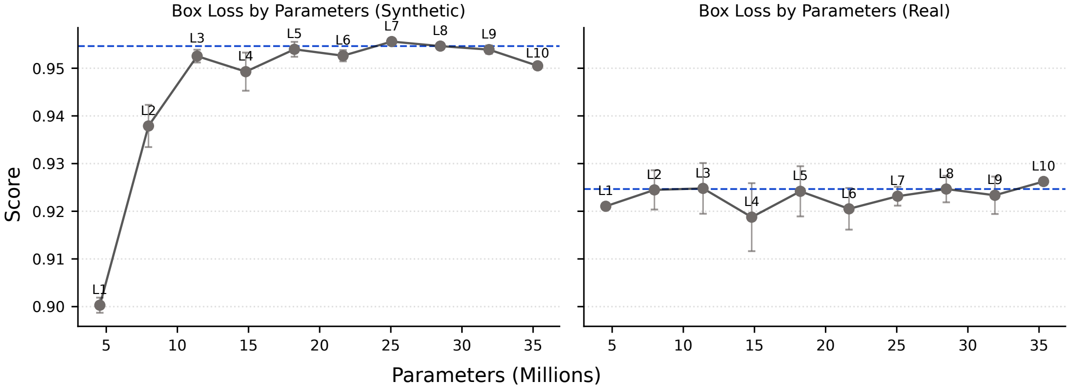

Figure S5.12: Box-Loss across the same dense encoder-depth sweep on synthetic and GENA data. Both datasets show a broad high-performing regime rather than a single isolated optimum, and the horizontal blue line marks the selected configuration used in the present study. Error bars indicate seed-to-seed variation where visible; points without visible error bars have near-zero deviation at the plotted scale.

S6 Example conversion: model outputs to peak-box evaluation

Text S6.1. Worked examples across difficulty levels

The following samples illustrate how region- and event-level outputs translate into peak boxes and normalized Box-Loss component scores (higher is better). Each sample is introduced by a short text summary that lists labeled and predicted boxes, interprets TP/FN/FP contributions, and contextualizes the resulting Box-Loss before showing the corresponding region- and box-level plots. For clarity, the vertical dashed lines show only the events that directly contribute to box placement, not all event probabilities in the signal; the text comparisons likewise focus on those contributing events even when non-contributing events have high confidence.

Sample 0c0734f4-058d-4009-a9e1-fd27a019eb7a

This sample shows three isolated peaks with labeled boxes (33–116), (168–215), and (234–250) and predicted boxes (33–116), (168–215), and (234–249); the only discrepancy is a one-index offset on the final endpoint. The normalized component scores are TP = 0.9730, FN = 1.0000, and FP = 1.0000, yielding a Box-Loss of 0.9952. The example schematically illustrates how peak boxes are placed for multiple standalone, low-amplitude peaks.

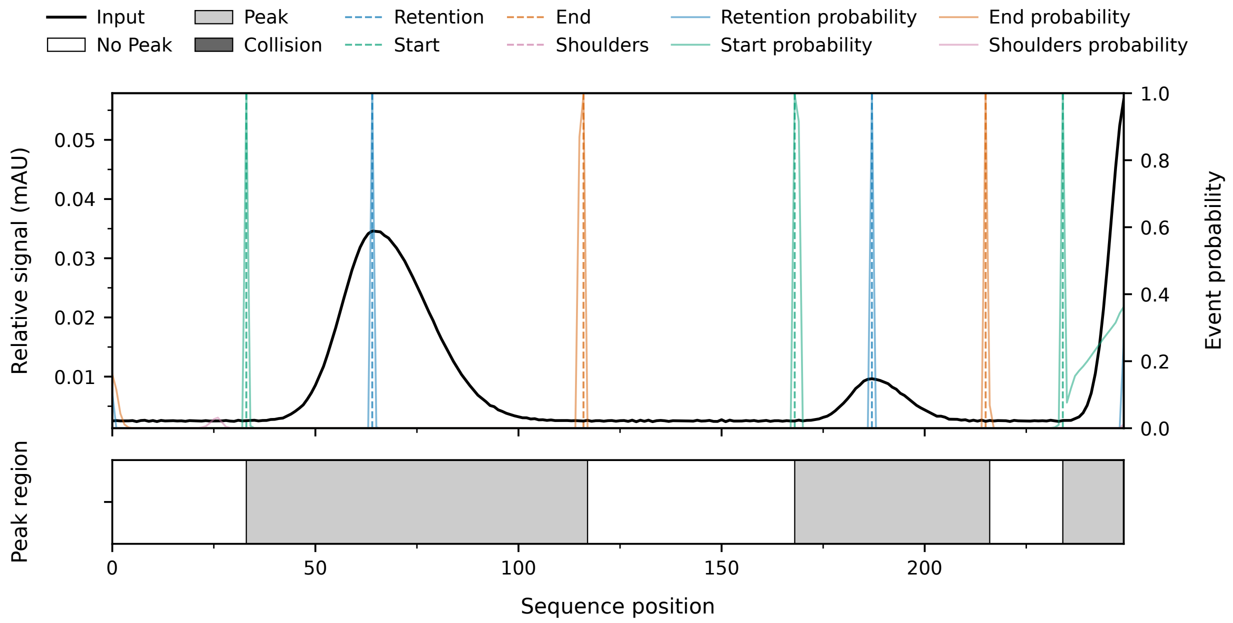

Figure S6.1: Peak event detection and region classification performance on a synthetic chromatogram (id 0c0734f4-058d-4009-a9e1-fd27a019eb7a) with multiple standalone peaks. Top: Input signal (solid black line) with color-coded event probabilities and reference labels shown as vertical dashed lines. The model accurately identifies retention (blue), start (green), and end (orange) positions with high probability scores. Bottom: Predicted region classification showing baseline (white), single-peak regions (light gray), and collision regions (dark gray) where multiple peaks are significant.

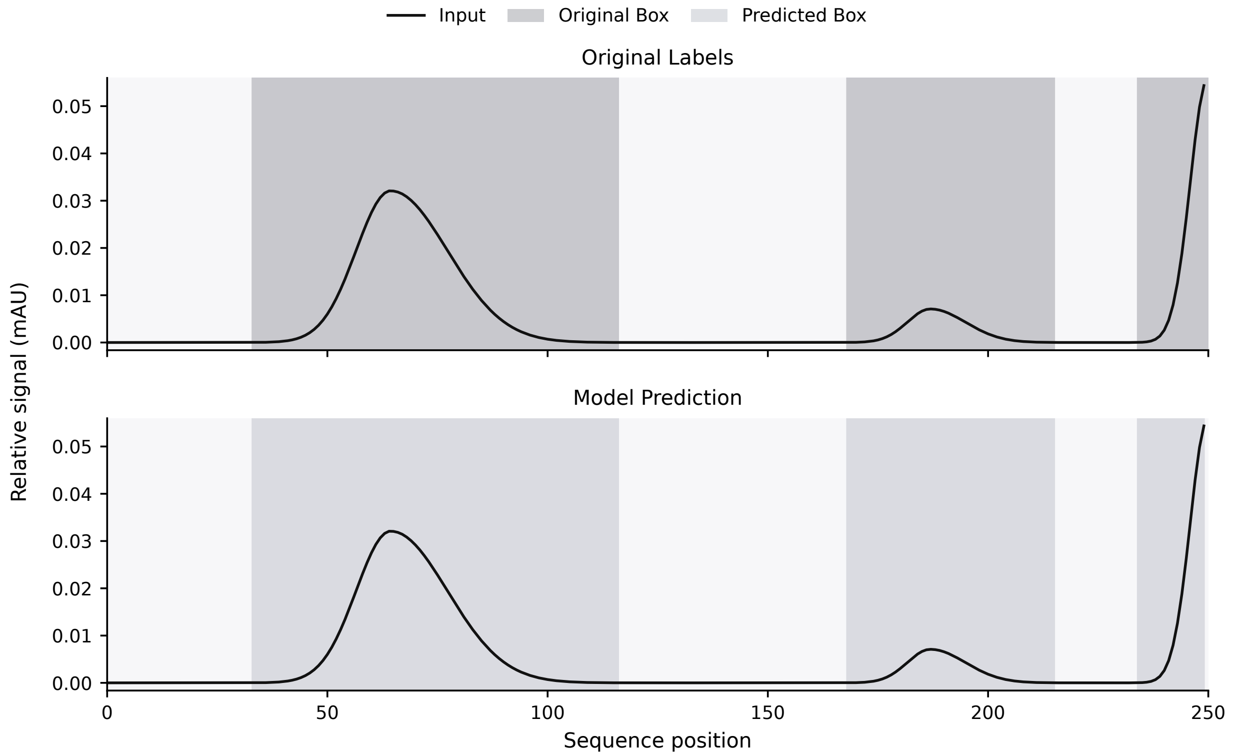

Figure S6.2: Comparison of reference and predicted boxes for a synthetic chromatogram. Top: Input signal with original box annotations (gray shaded areas). Bottom: Same signal with predicted boxes (light gray areas). The predicted boxes closely match the reference, consistent with the near-perfect Box-Loss of 0.9952.

Sample 98ecbebb-27dc-4c08-8c39-6f485f31461d

This example contains two colliding peaks, where a smaller peak interacts with a larger peak and forms a shoulder. Labeled boxes (108–135) and (136–189) are matched by predicted boxes (108–135) and (136–188), with a one-index offset on the right endpoint. The normalized component scores are TP = 0.9672, FN = 1.0000, and FP = 1.0000, yielding a Box-Loss of 0.9942. The box plot highlights that the split point is not placed at the valley minimum but nearer the midpoint between valley and right-peak apex (position 135), reflecting the intended deviation from a strict valley-to-valley convention.

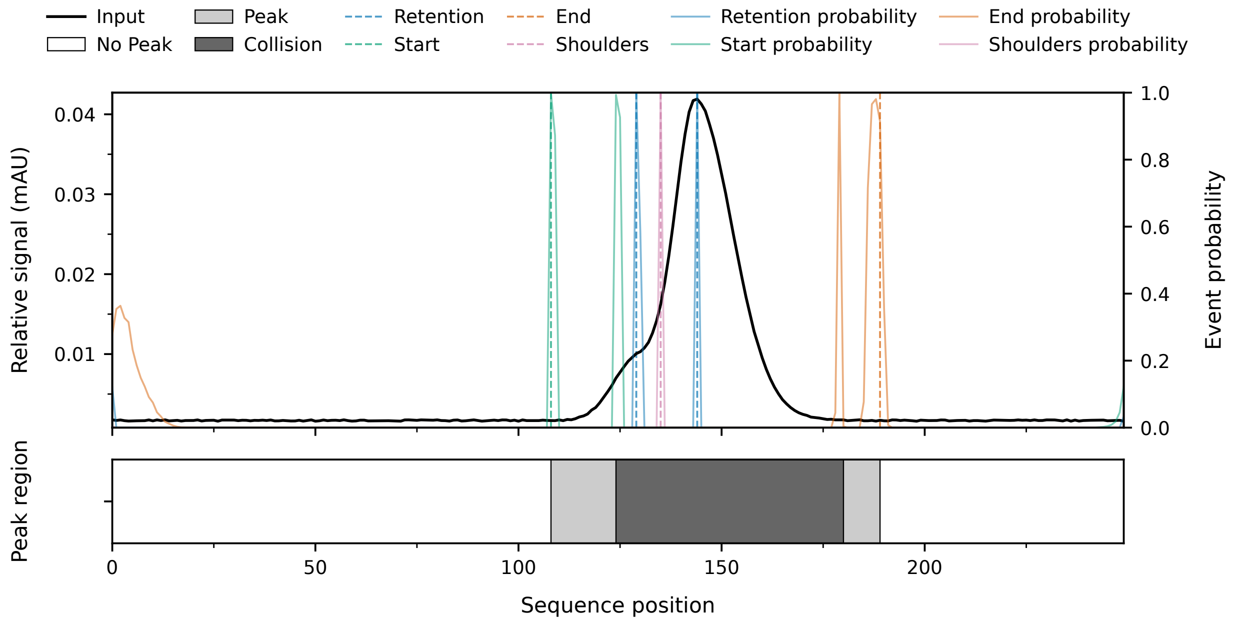

Figure S6.3: Peak event detection and region classification performance on a synthetic chromatogram (id 98ecbebb-27dc-4c08-8c39-6f485f31461d) with two interacting peaks and a shoulder. Top: Input signal (solid black line) with color-coded event probabilities and reference labels shown as vertical dashed lines. Bottom: Predicted region classification showing baseline (white), single-peak regions (light gray), and collision regions (dark gray) where multiple peaks are significant.

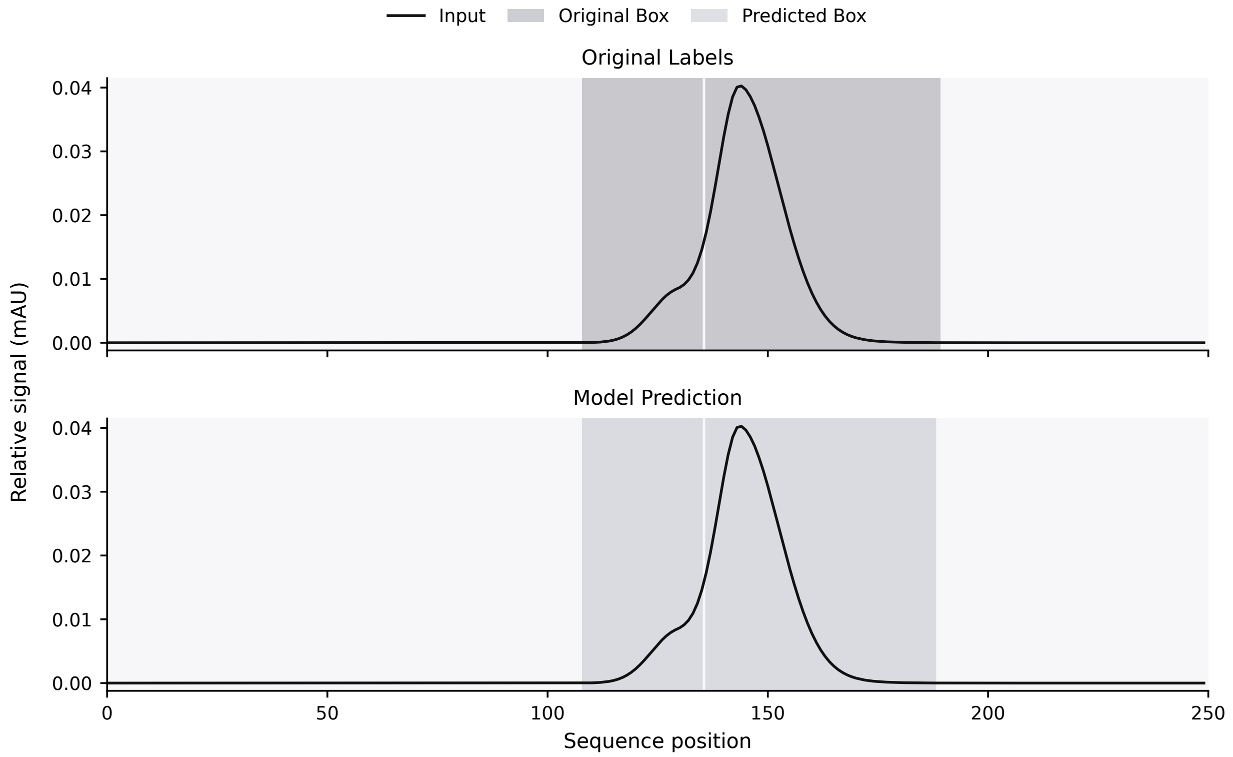

Figure S6.4: Comparison of reference and predicted boxes for a synthetic chromatogram. Top: Input signal with original box annotations (gray shaded areas). Bottom: Same signal with predicted boxes (light gray areas). The split point near 135 sits between the valley and the right-peak apex, illustrating the shoulder-aware boundary rule and the resulting near-perfect Box-Loss of 0.9942.

Sample 18fb6636-7600-4d09-8f69-4dd8b0b75780

This example contains multiple interacting peaks, and the model separates the overlaps with high accuracy. Labeled boxes are (window start–51), (52–109), (110–140), and (232–window end), matched by predicted boxes (29–51), (52–108), (109–141), and (232–249). The normalized component scores are TP = 0.7341, FN = 1.0000, and FP = 1.0000, yielding a Box-Loss of 0.9531. A common edge effect is visible at the left boundary: the model ends the leftmost box too early as the signal transitions into the baseline, while the rightmost large peak is localized accurately.

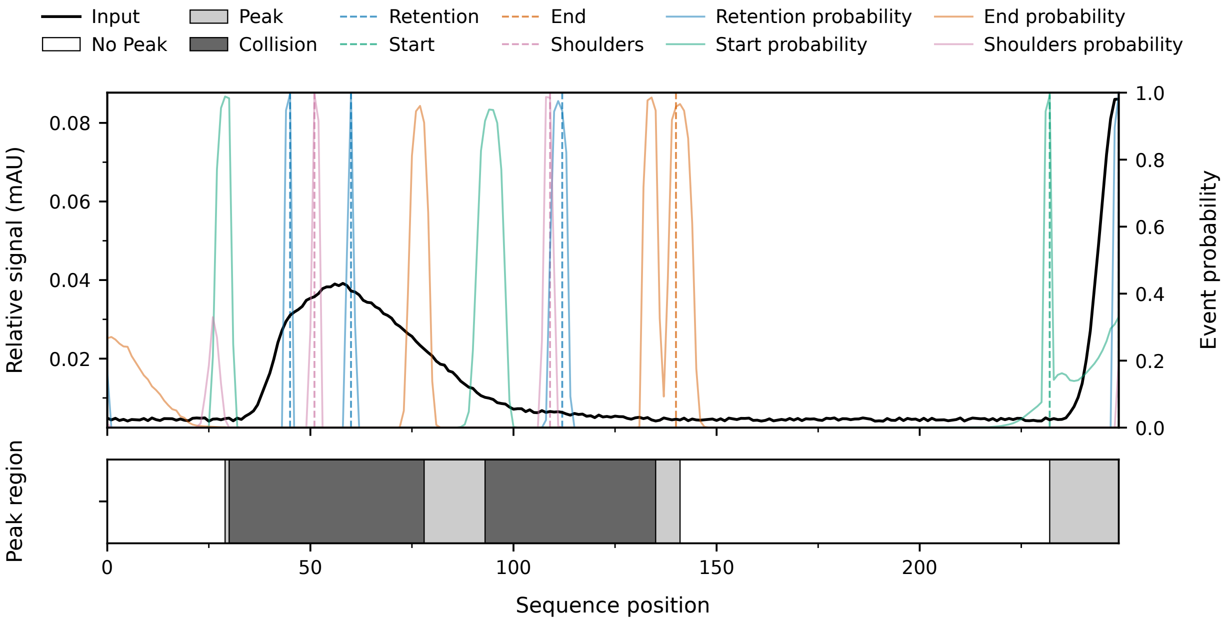

Figure S6.5: Peak event detection and region classification performance on a synthetic chromatogram (id 18fb6636-7600-4d09-8f69-4dd8b0b75780) with multiple interacting peaks. Top: Input signal (solid black line) with color-coded event probabilities and reference labels shown as vertical dashed lines. Bottom: Predicted region classification showing baseline (white), single-peak regions (light gray), and collision regions (dark gray) where multiple peaks are significant.

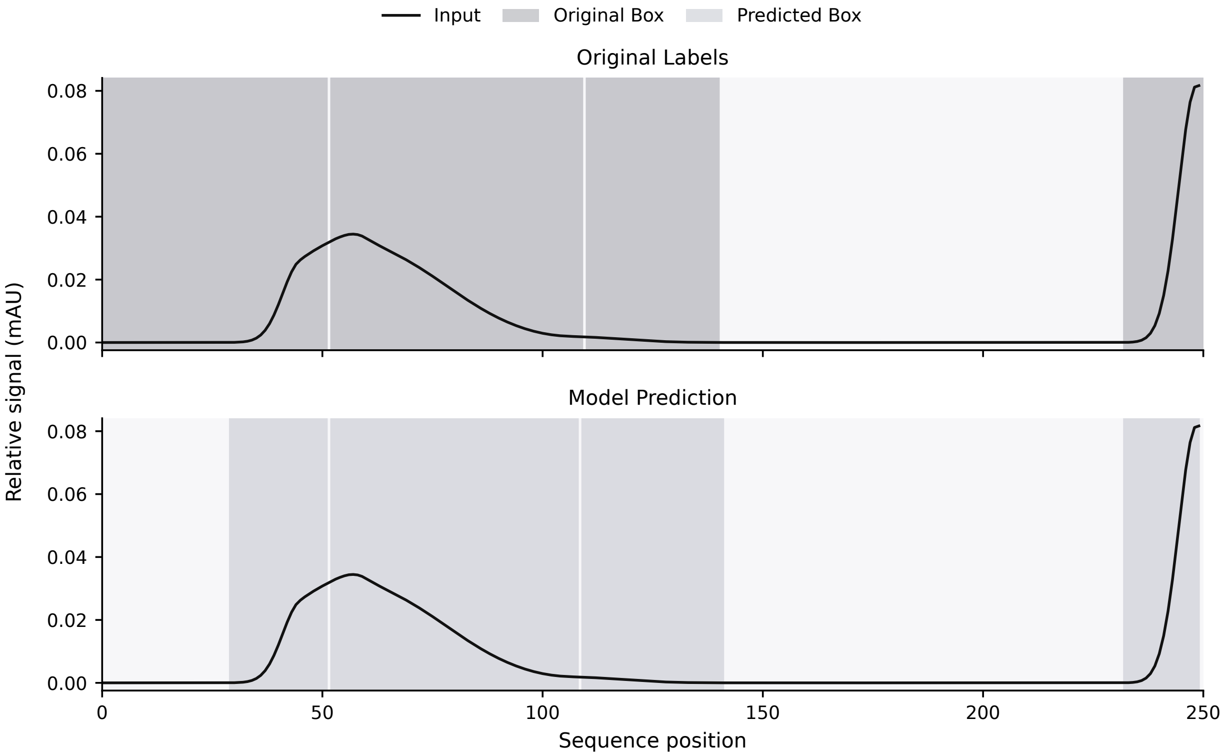

Figure S6.6: Comparison of reference and predicted boxes for a synthetic chromatogram. Top: Input signal with original box annotations (gray shaded areas). Bottom: Same signal with predicted boxes (light gray areas). The split points track the intended shoulder-aware boundaries, while the leftmost box ends slightly early at the baseline transition.

Sample cdc916bd-4c82-4131-b3da-11e4082f17b7

This sample contains very flat, broad peaks that are difficult to separate from a drifting baseline. Labeled boxes (0–36), (37–81), (82–165), (166–195), (196–219), and (241–250) are compared to predicted boxes (0–36), (37–81), (82–165), (166–195), and (196–220). The normalized component scores are TP = 0.9579, FN = 0.8778, and FP = 1.0000, yielding a Box-Loss of 0.8927. The prediction captures the peak landscape well, but repeated edge truncations indicate that the hard-coded aggregation rules fail to fully translate the model's boundary signals near the margins.

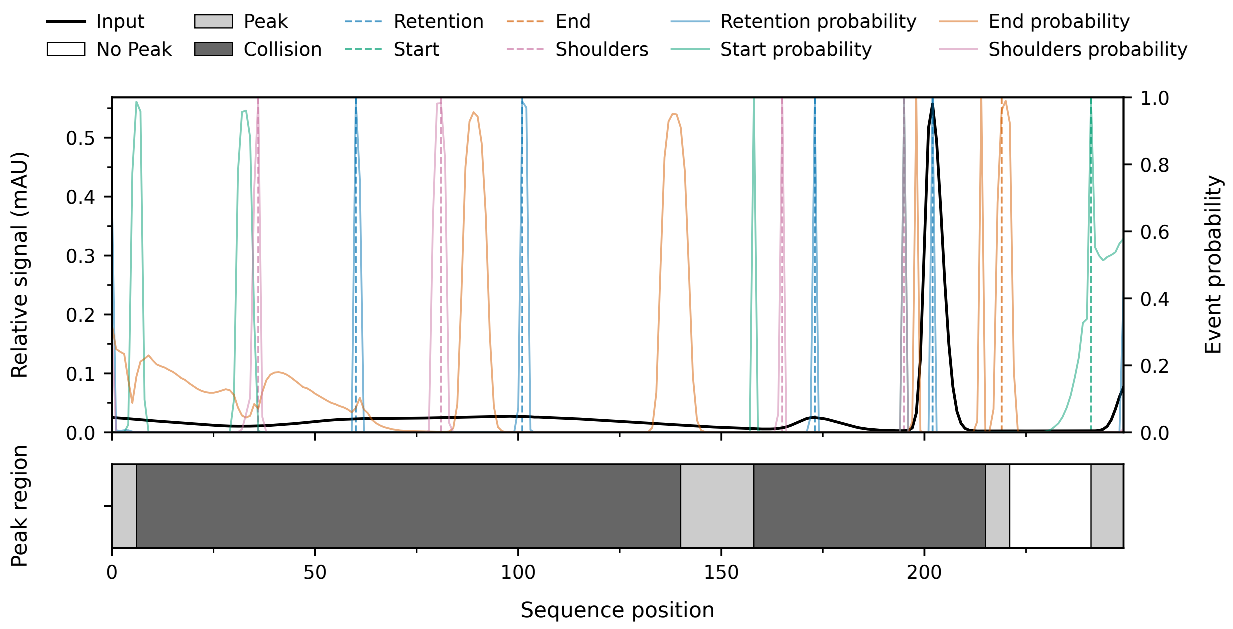

Figure S6.7: Peak event detection and region classification performance on a synthetic chromatogram (id cdc916bd-4c82-4131-b3da-11e4082f17b7) with broad, low-amplitude peaks. Top: Input signal (solid black line) with color-coded event probabilities and reference labels shown as vertical dashed lines. Bottom: Predicted region classification showing baseline (white), single-peak regions (light gray), and collision regions (dark gray) where multiple peaks are significant.

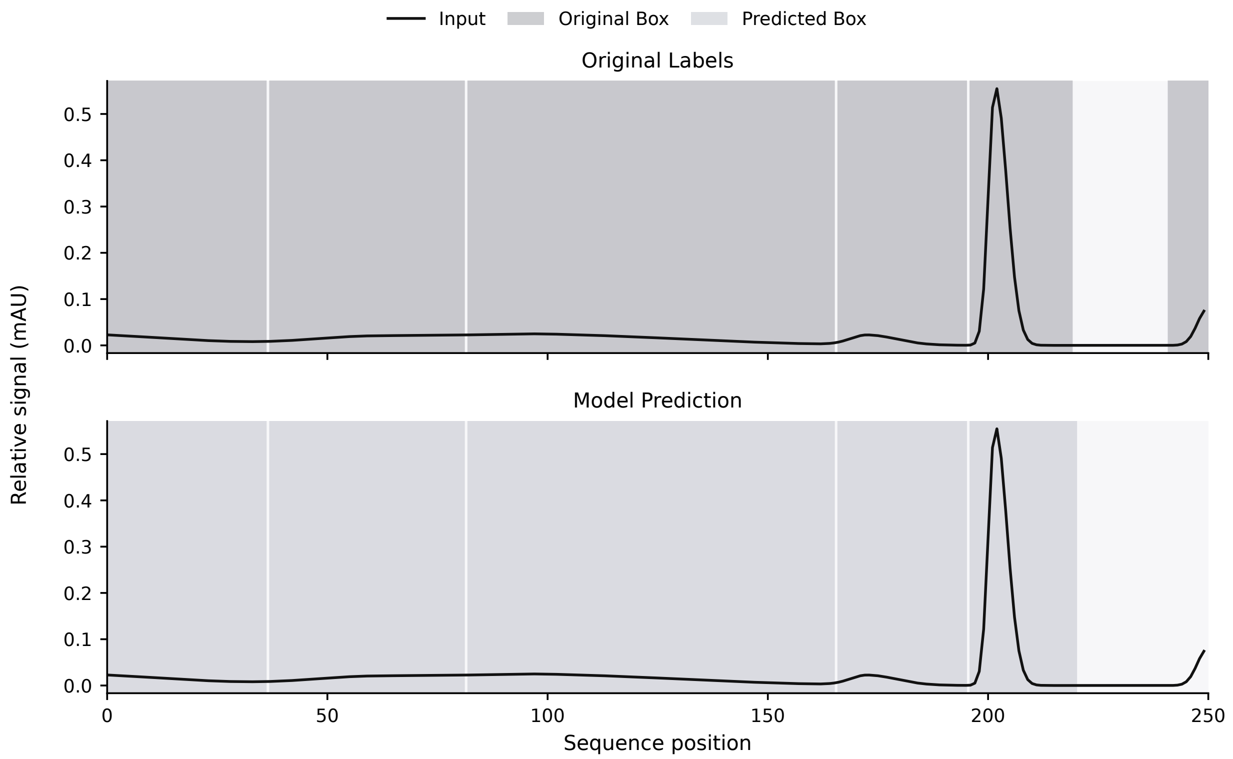

Figure S6.8: Comparison of reference and predicted boxes for a synthetic chromatogram. Top: Input signal with original box annotations (gray shaded areas). Bottom: Same signal with predicted boxes (light gray areas). The model resolves the broad peaks, while aggregation near the boundaries truncates the outermost intervals.

Sample 32ef5635-210a-4c2a-99c4-7c8a685b4862

This sample follows the same pattern as the earlier examples. The main peak is over-split, with the tail-facing region deviating from the labeled boundary, and the right edge shows an incorrect split near position 230. The event plot indicates a detected shoulder, but strong probability fluctuations close to the window boundary lead to a missed aggregation step. The resulting Box-Loss is 0.868351.

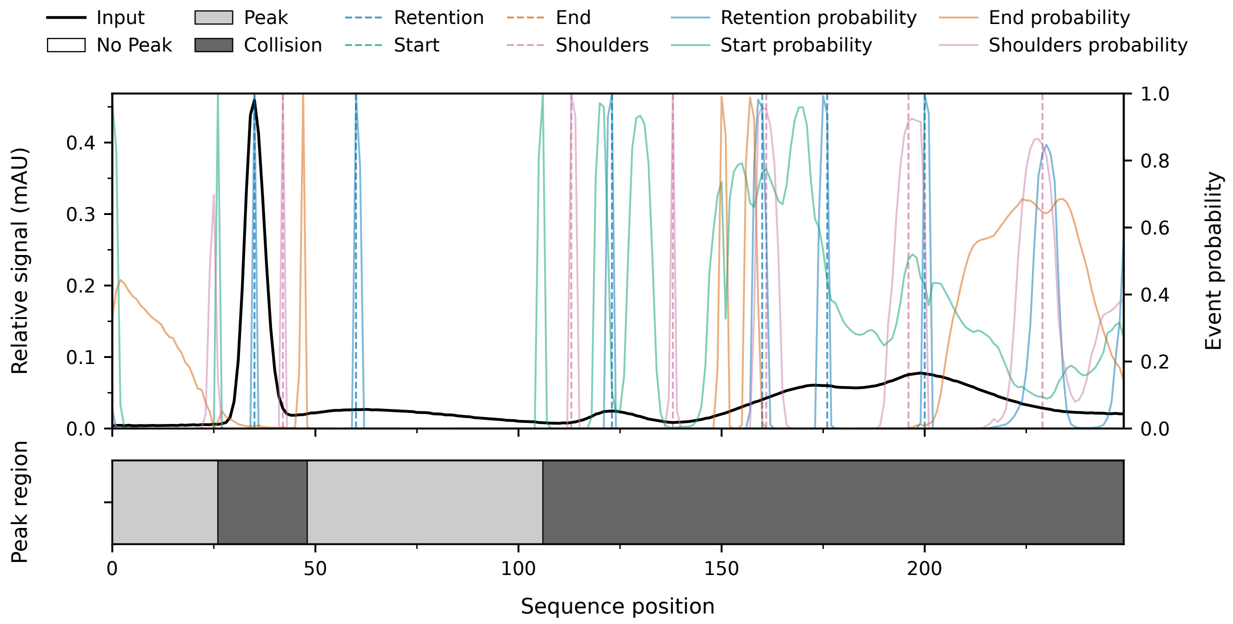

Figure S6.9: Peak event detection and region classification performance on a synthetic chromatogram (id 32ef5635-210a-4c2a-99c4-7c8a685b4862). Top: Input signal (solid black line) with color-coded event probabilities and reference labels shown as vertical dashed lines. Bottom: Predicted region classification showing baseline (white), single-peak regions (light gray), and collision regions (dark gray) where multiple peaks are significant.

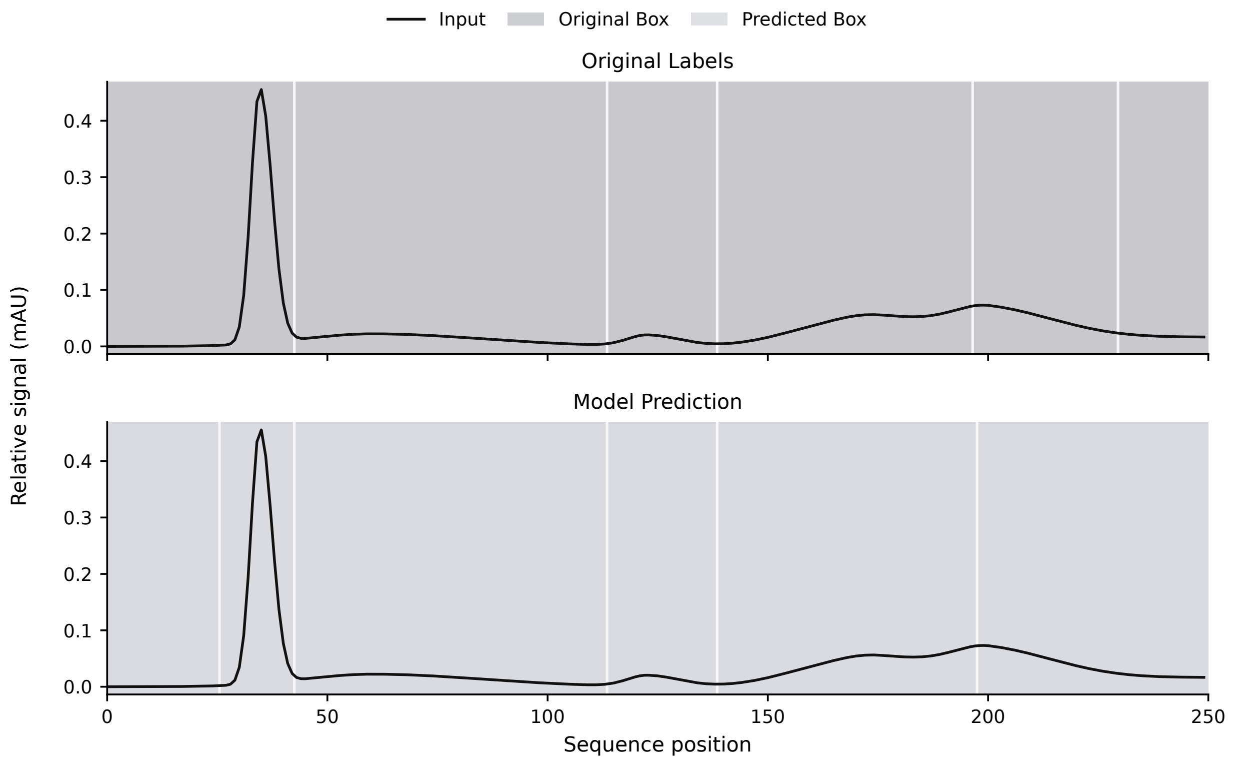

Figure S6.10: Comparison of reference and predicted boxes for a synthetic chromatogram. Top: Input signal with original box annotations (gray shaded areas). Bottom: Same signal with predicted boxes (light gray areas).

Sample 71c6b483-43d5-476a-a13c-9948bda75381

This example highlights two recurring patterns. First, at around position 100, two labeled boxes (74–102) and (103–114) are not separated by the prediction, which instead keeps a single interval (74–114). Second, the main peak is split into multiple boxes, reflecting a tendency to over-segment large, mildly asymmetric peaks where analysts would typically keep a single interval unless clear local morphology supports a split. Labeled boxes (0–73), (74–102), (103–114), (115–149), (150–178), (179–211), (212–240), and (241–250) are compared to predicted boxes (0–73), (74–114), (115–125), (126–149), (150–178), (179–211), (212–240), and (241–250). The normalized component scores are TP = 0.5919, FN = 1.0000, and FP = 1.0000, yielding a Box-Loss of 0.9280.

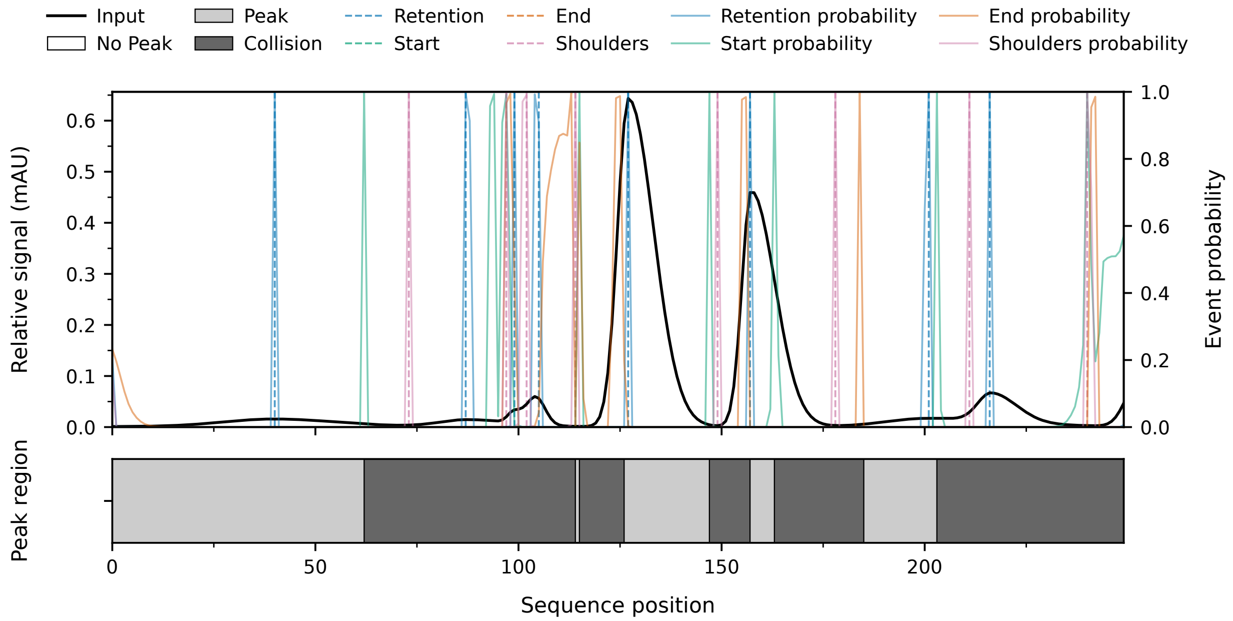

Figure S6.11: Peak event detection and region classification performance on a synthetic chromatogram (id 71c6b483-43d5-476a-a13c-9948bda75381) with a dominant peak and several smaller components. Top: Input signal (solid black line) with color-coded event probabilities and reference labels shown as vertical dashed lines. Bottom: Predicted region classification showing baseline (white), single-peak regions (light gray), and collision regions (dark gray) where multiple peaks are significant.

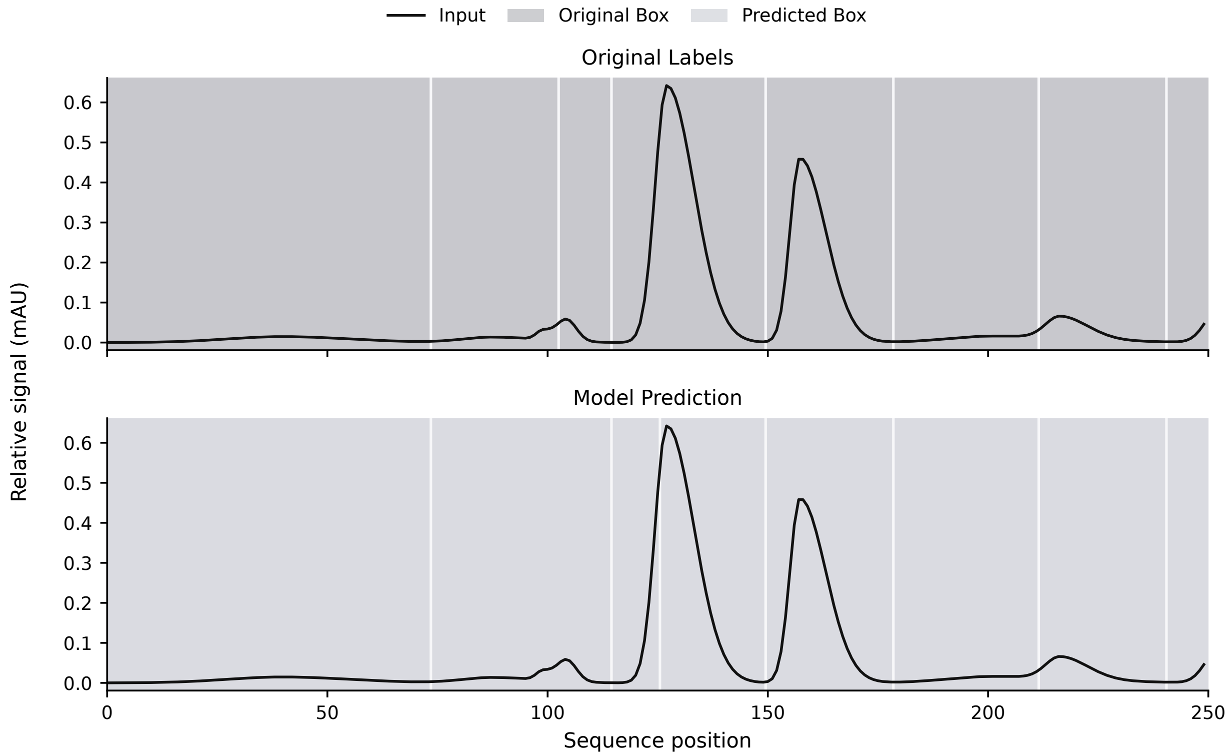

Figure S6.12: Comparison of reference and predicted boxes for a synthetic chromatogram. Top: Input signal with original box annotations (gray shaded areas). Bottom: Same signal with predicted boxes (light gray areas). The merged boxes near position 100 and the split main peak illustrate the two dominant error modes described in the text.

Sample 3d50e5b8-b8a7-4d8d-ab0c-8444124f6fa8

This sample contains only noise with no peaks. A fraction of the training windows are intentionally peak-free to ensure the model learns to handle sliding windows that contain only background fluctuations.

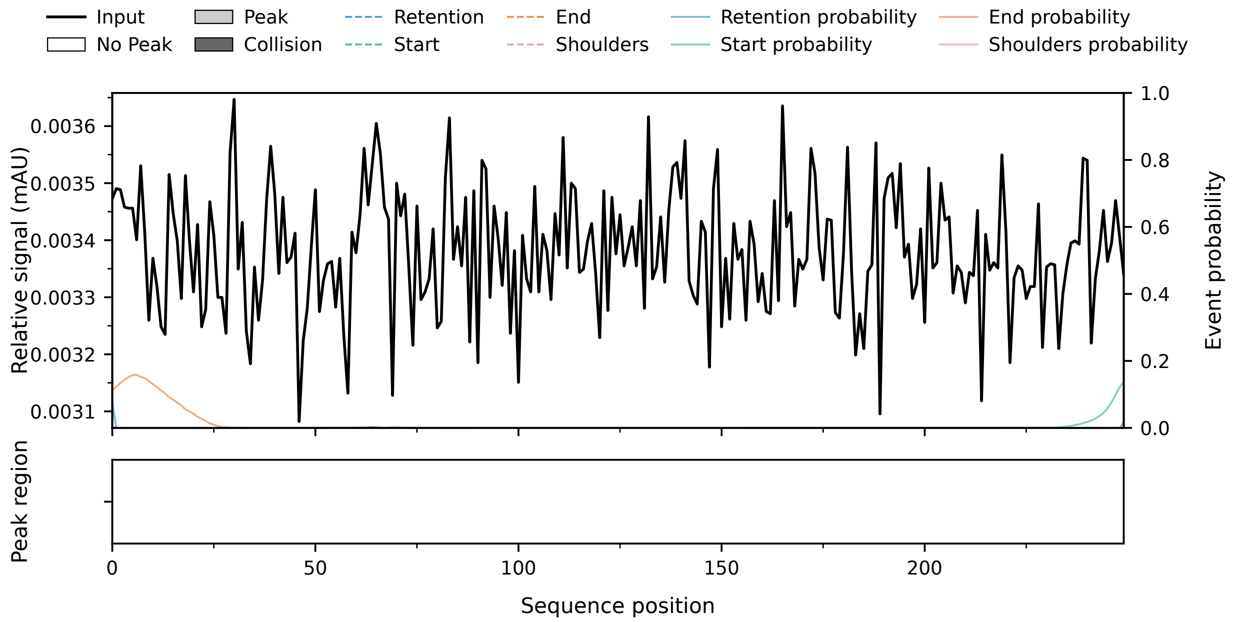

Figure S6.13: Peak event detection and region classification performance on a synthetic chromatogram (id 3d50e5b8-b8a7-4d8d-ab0c-8444124f6fa8) containing only background noise. Top: Input signal (solid black line) with color-coded event probabilities and reference labels shown as vertical dashed lines. Bottom: Predicted region classification showing baseline (white) without peak or collision regions.

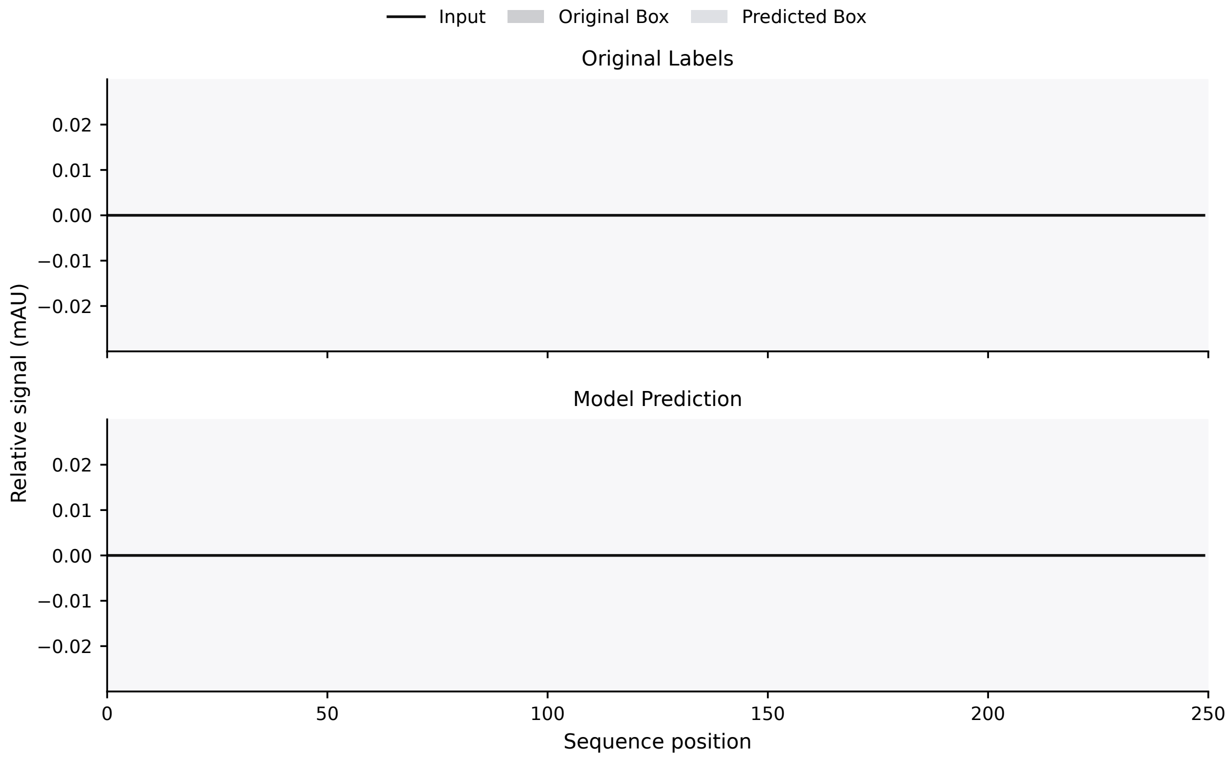

Figure S6.14: Comparison of reference and predicted boxes for a synthetic chromatogram. Top: Input signal with original box annotations (gray shaded areas). Bottom: Same signal with predicted boxes (light gray areas).

S7 Example conversion on real chromatograms

Text S7.1. Worked examples on experimental SEC traces

The following examples mirror the synthetic worked cases, but use full-length experimental chromatograms. Each sample includes a short summary of labeled and predicted boxes, TP/FN/FP contributions, and the resulting Box-Loss, followed by the corresponding chromatogram and box overlays.

Sample 416747c1-4308-4e40-9249-ec0c8dbcf465

This real SEC chromatogram contains a dominant main peak and a distinct light molecular weight (LMW) peak that forms a shoulder around position 300. In single-peak regions, event probabilities are narrow and well localized, supporting straightforward post-processing. In collision regions, start/end probabilities broaden and show lower precision while retaining high recall, whereas retention and shoulder events remain sharply resolved. The box plot illustrates how these events map into the box logic described above. Overall, the integration is consistent but the smaller side-product boundaries are slightly wider than the reference, and additional peaks are annotated. This reflects routine process limits on the maximum number of annotated peaks, which can under-report minor features. In this trace, extra peak groups appear both before and after the main peak region. The resulting normalized Box-Loss is 0.9542.

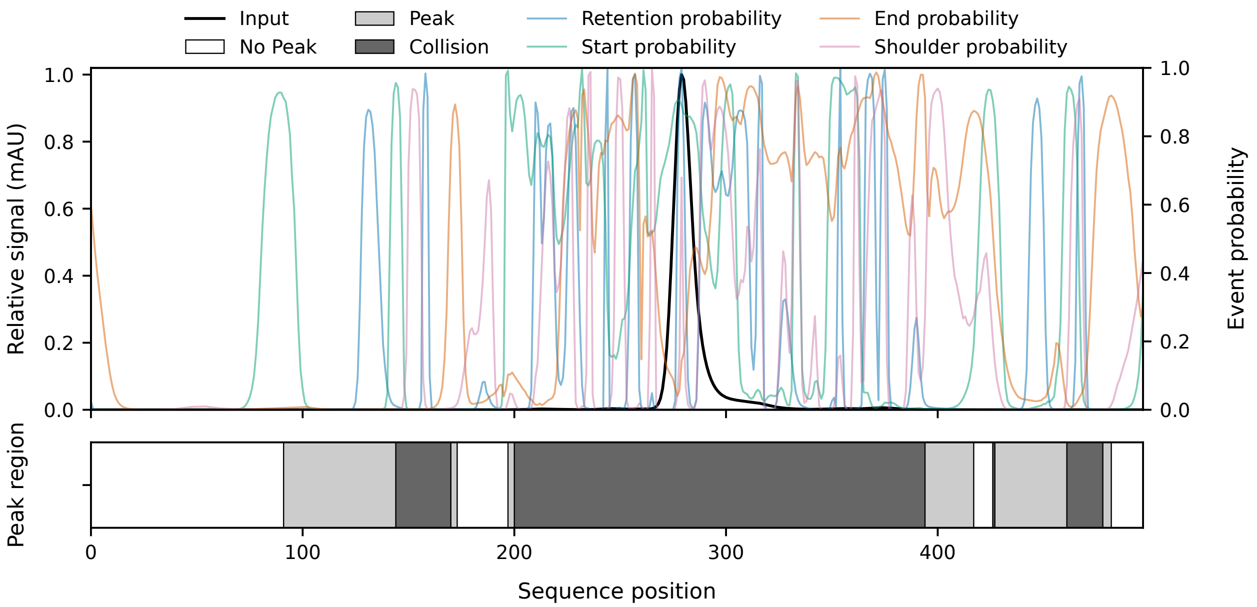

Figure S7.1: Peak event detection and region classification performance on a real SEC chromatogram (id 416747c1-4308-4e40-9249-ec0c8dbcf465) with a dominant main peak and several side-product peaks. Top: Input signal (solid black line) with color-coded event probabilities and reference labels shown as vertical dashed lines. Bottom: Predicted region classification showing baseline (white), single-peak regions (light gray), and collision regions (dark gray) where multiple peaks are significant.

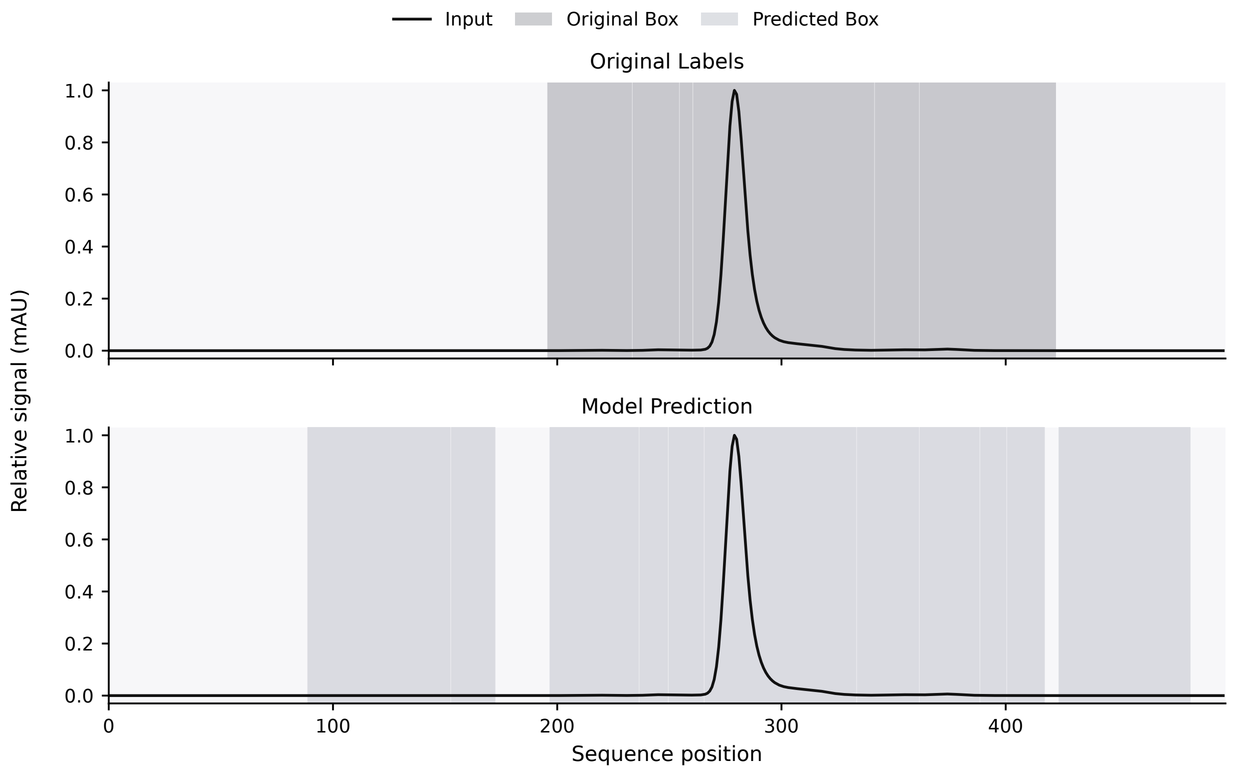

Figure S7.2: Comparison of reference and predicted boxes for a real SEC chromatogram. Top: Input signal with original box annotations (gray shaded areas). Bottom: Same signal with predicted boxes (light gray areas). The normalized Box-Loss is 0.9542.

Sample c497f514-19f3-4350-abdc-d683728bcfba

This real SEC chromatogram shows a pronounced main peak with multiple side-product peaks. The predicted boxes largely match the labeled regions, but the model annotates additional peaks and subdivides the main-peak neighborhood into finer intervals. The resulting normalized Box-Loss is 0.9432.

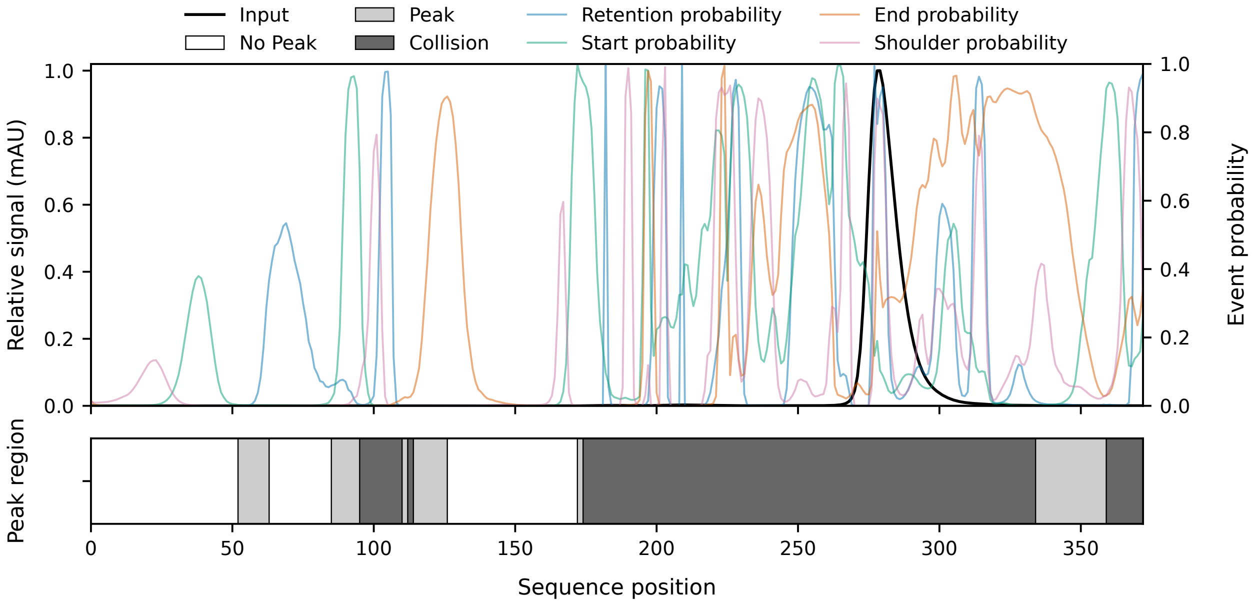

Figure S7.3: Peak event detection and region classification performance on a real SEC chromatogram (id c497f514-19f3-4350-abdc-d683728bcfba) with a dominant main peak and side-product features. Top: Input signal (solid black line) with color-coded event probabilities and reference labels shown as vertical dashed lines. Bottom: Predicted region classification showing baseline (white), single-peak regions (light gray), and collision regions (dark gray) where multiple peaks are significant.

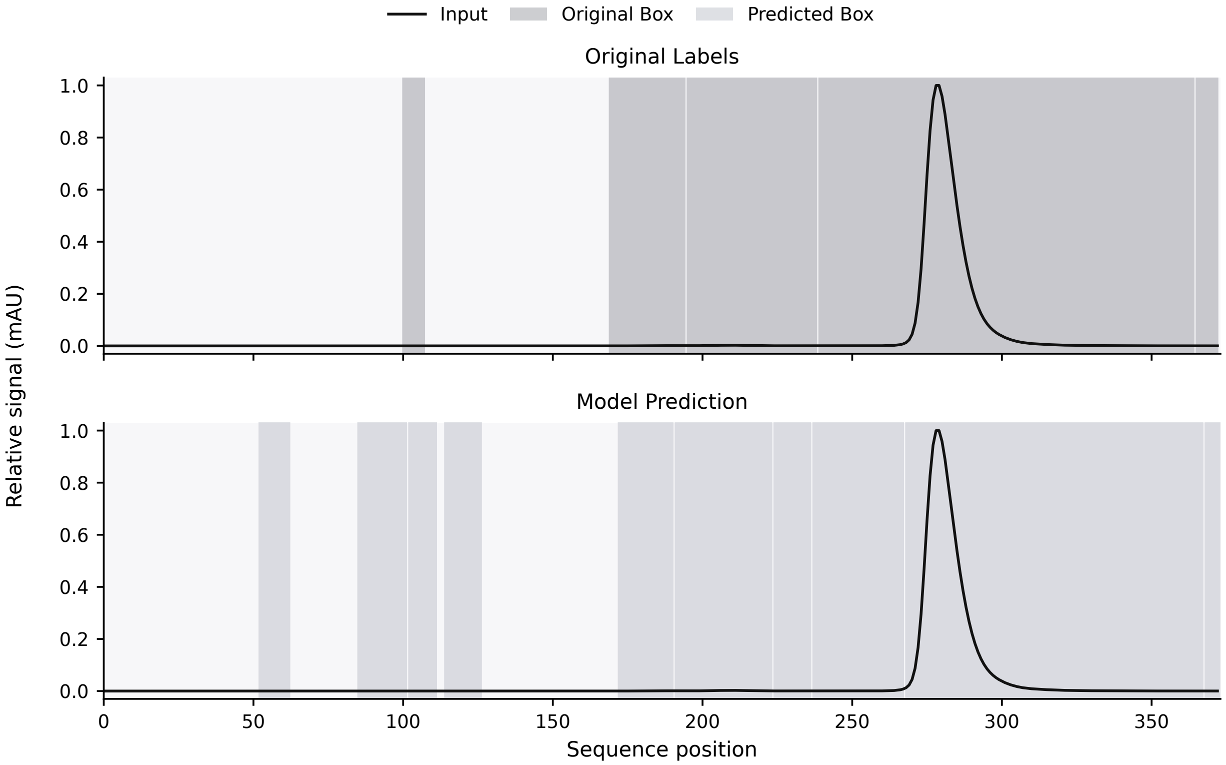

Figure S7.4: Comparison of reference and predicted boxes for a real SEC chromatogram. Top: Input signal with original box annotations (gray shaded areas). Bottom: Same signal with predicted boxes (light gray areas). The normalized Box-Loss is 0.9432.

Sample 9ed315fb-5c5f-4d52-8eeb-5939bd1c7791

This sample again contains a pronounced main peak, now with a clearer high molecular weight (HMW) peak preceding the main peak. The prediction splits the HMW and main regions, but also infers a broad shoulder region beyond position 300 where event probabilities are diffuse and imprecise, producing a large undifferentiated box. This contributes to the largest deviation from the human annotation in this set. The resulting normalized Box-Loss is 0.8580.

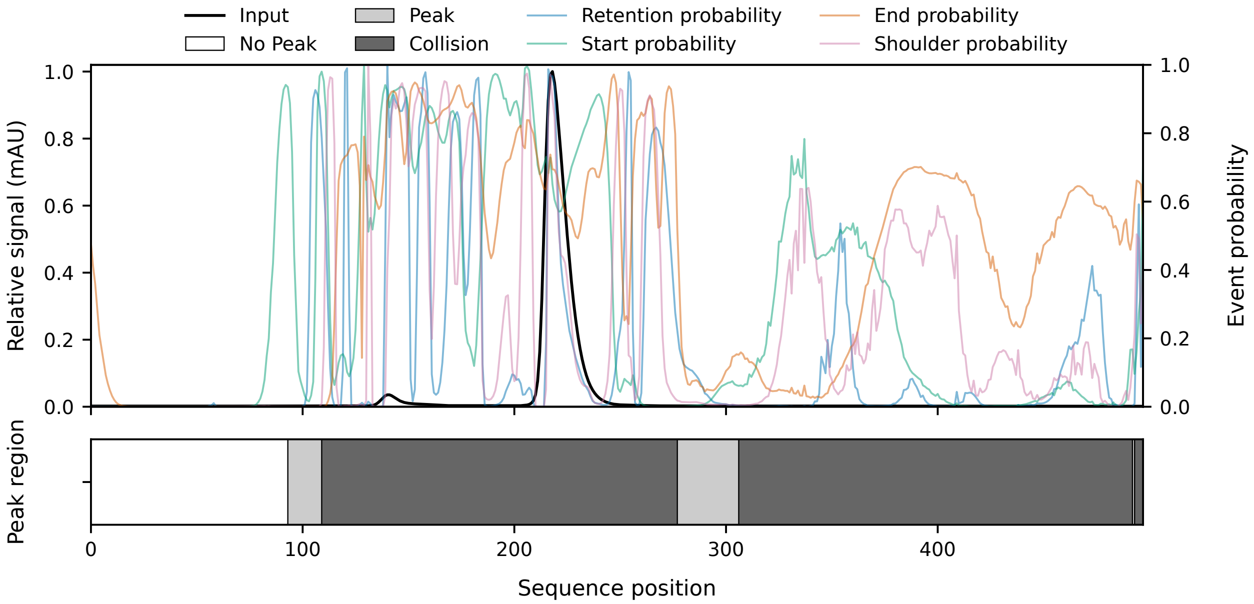

Figure S7.5: Peak event detection and region classification performance on a real SEC chromatogram (id 9ed315fb-5c5f-4d52-8eeb-5939bd1c7791) with a dominant main peak and a visible HMW peak. Top: Input signal (solid black line) with color-coded event probabilities and reference labels shown as vertical dashed lines. Bottom: Predicted region classification showing baseline (white), single-peak regions (light gray), and collision regions (dark gray) where multiple peaks are significant.

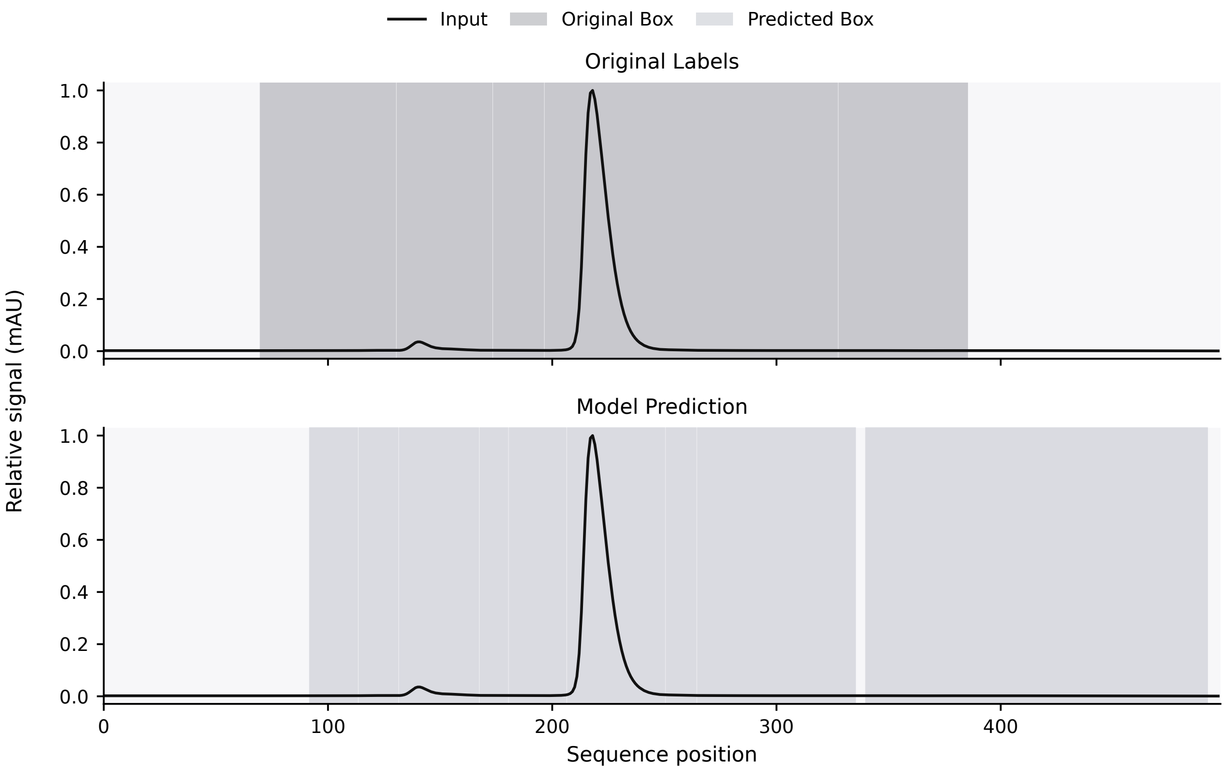

Figure S7.6: Comparison of reference and predicted boxes for a real SEC chromatogram. Top: Input signal with original box annotations (gray shaded areas). Bottom: Same signal with predicted boxes (light gray areas). The normalized Box-Loss is 0.8580.
